# Supplementary material for: Critical research gaps in treating growth faltering in infants under 6 months: A systematic review and meta-analysis
Source: PLOS Glob Public Health. 2024 Jan 8;4(1):e0001860. doi: 10.1371/journal.pgph.0001860 (PMC10773941; doi:10.1371/journal.pgph.0001860)
Supplement: S1 Appendix — (PDF) [file pgph.0001860.s002.pdf]

Appendix 1. Search Strategy

Search number	Query
10	#8 AND #9
9	((("english"[Language]) OR ("spanish"[Language])) OR ("chinese"[Language])) OR ("french"[Language])
8	#4 AND #7
7	#5 OR #6
6	"quasiexperiment*" [tiab] OR "quasi experiment*" [tiab] OR "exogenous variation*" [tiab] OR "natural experiment*" [tiab] OR "Matched controls" [tiab] OR "Counterfactual outcome*" [tiab] OR "Rubin causal model*" [tiab] OR "potential outcomes model*" [tiab] OR (Identification [tiab] AND (strategy [tiab] OR assumptions [tiab] OR conditions [tiab])) OR (conditional [tiab] AND (independence [tiab] OR ignorability [tiab])) OR "unobserved heterogeneity" [tiab] OR Unconfoundness [tiab] OR Confounding [tiab] OR ("instrumental variable*" [tiab] AND (analysis [tiab] OR analyses [tiab] OR estimation [tiab])) OR "overidentification" [tiab] OR "overidentifying" [tiab] OR "regression discontinuity analys*" [tiab] OR ((balancing [tiab] OR imbalance [tiab] OR balanced [tiab] OR imbalanced [tiab]) AND covariates [tiab]) OR "interrupted time series" [tiab] OR "difference studies" [tiab] OR (controlled [tiab] AND before [tiab] AND after [tiab]) OR ((exact [tiab] OR score [tiab] OR genetic [tiab] OR "nearest neighbor" [tiab] OR "nearest neighbour" [tiab] OR caliper [tiab] OR radius [tiab] OR "kernel density" [tiab] OR blocking [tiab] OR "stratification of interval" [tiab]) AND matching [tiab]) OR ("Inverse probability weight*" [tiab] AND "estimat*" [tiab]) OR ("doubly robust" [tiab] AND (regression [tiab] OR estimate* [tiab])) OR ((treatment [tiab] OR switching [tiab] OR selection [tiab] OR selectivity [tiab]) AND regression [tiab]) OR "selection model" [tiab] OR "selectivity model" [tiab] OR "heckit model" [tiab] OR "heckman sample selection" [tiab] OR "selection correction" [tiab] OR "two stage residual inclusion" [tiab] OR "regression discontinuity" [tiab] OR "sharp design" [tiab] OR "fuzzy design" [tiab] OR "Forcing variable*" [tiab] OR (difference* [tiab] AND difference* [tiab]) OR (change* [tiab] AND change* [tiab]) OR "Fixed effects and panel data" [tiab] OR "full information maximum likelihood" [tiab] OR ((health [tiab] OR economic [tiab]) AND shock* [tiab]) OR "natural controls" [tiab]
5	((randomized controlled trial[pt]) OR (controlled clinical trial[pt]) OR (randomized[tiab] OR randomised[tiab]) OR (placebo[tiab]) OR (drug therapy[sh]) OR (randomly[tiab]) OR (trial[tiab]) OR (groups[tiab]))
4	#1 AND #2 AND #3

3	"Bottle Feeding"[Mesh] OR "Breast Feeding"[Mesh] OR "Milk, Human"[Mesh] OR "Milk Banks"[Mesh] OR "Lactation"[Mesh] OR "Weaning"[Mesh] OR "Infant Formula"[Mesh] OR "Milk Substitutes"[Mesh] OR "bottle feeding" [tiab] OR "breast milk" [tiab] OR "breast milk fortifier" [tiab] OR "breast milk substitute" [tiab] OR "breastmilk" [tiab] OR "complementary food" [tiab] OR "cup fed" [tiab] OR "cup feed*" [tiab] OR "early weaning" [tiab] OR "establishing breastfeeding" [tiab] OR "human milk" [tiab] OR "infant feeding practices" [tiab] OR "infant formula" [tiab] OR "spoon fed" [tiab] OR "spoon feed*" [tiab] OR "supplementary suck*" [tiab] OR "supplementary food" [tiab] OR "Donor milk" [tiab] OR "Donor breast milk" [tiab] OR "Donated breast milk" [tiab] OR "Milk bank" [tiab] OR "wet nurse*" [tiab] OR "relact*" [tiab] OR "milk sharing" [tiab] OR "breast fed" [tiab] OR "f-75" [tiab] OR "f-100" [tiab]
2	"Severe Acute Malnutrition"[Mesh] OR "Malnutrition"[Mesh] OR "Edema"[Mesh] OR "Failure to Thrive"[Mesh] OR "Fetal Growth Retardation"[Mesh] OR "Growth Disorders"[Mesh] OR "acute malnutrition" [tiab] OR "bilateral edema" [tiab] OR "bilateral oedema" [tiab] OR "bilateral pitting edema" [tiab] OR "bilateral pitting oedema" [tiab] OR "edema" [tiab] OR "edematous" [tiab] OR "failure to thrive" [tiab] OR "fetal growth restriction" [tiab] OR "fetal growth retardation" [tiab] OR "foetal growth restriction" [tiab] OR "foetal growth retardation" [tiab] OR "FTT" [tiab] OR "growth disorder" [tiab] OR "growth faltering" [tiab] OR "intrauterine growth restriction" [tiab] OR "intrauterine growth retardation" [tiab] OR "IUGR" [tiab] OR "kwashiorkor" [tiab] OR "malnourish" [tiab] OR "malnutrition" [tiab] OR "marasmus" [tiab] OR "mid upper arm circumference" [tiab] OR "mid-upper arm circumference" [tiab] OR "MUAC" [tiab] OR "oedema" [tiab] OR "oedematous" [tiab] OR "PEM" [tiab] OR "SAM" [tiab] OR "severe acute malnutrition" [tiab] OR "severe malnutrition" [tiab] OR "starv*" [tiab] OR "stunt*" [tiab] OR "undernourished" [tiab] OR "undernutrition" [tiab] OR "wast*" [tiab] OR "wasted" [tiab] OR "wasted" [tiab] OR "wasting" [tiab] OR "wasting" [tiab] OR "weight for age" [tiab] OR "weight for height" [tiab] OR "weight for length" [tiab] OR "weight-for-age" [tiab] OR "weight-for-height" [tiab] OR "weight-for-length" [tiab]
1	"Infant"[Mesh] OR "early life" [tiab] OR "infant*" [tiab] OR "infants under 6 months" [tiab] OR "LBW" [tiab] OR "low birth weight" [tiab] OR "low birthweight" [tiab] OR "low-birth-weight" [tiab] OR "neonat*" [tiab] OR "neonate" [tiab] OR "neonates" [tiab] OR "newborn" [tiab] OR "newborns" [tiab] OR "premature" [tiab] OR "SGA" [tiab] OR "small for gestational age" [tiab] OR "small-for-gestational-age" [tiab] OR "pre-mature" [tiab] OR "very low birth weight" [tiab] OR "very low birthweight" [tiab] OR "VLBW" [tiab] OR "preterm" [tiab] OR "pre-term" [tiab]
