# Supplementary material for: Critical research gaps in treating growth faltering in infants under 6 months: A systematic review and meta-analysis
Source: PLOS Glob Public Health. 2024 Jan 8;4(1):e0001860. doi: 10.1371/journal.pgph.0001860 (PMC10773941; doi:10.1371/journal.pgph.0001860)
Supplement: S2 Appendix — (PDF) [file pgph.0001860.s003.pdf]

Appendix 2. Excluded studies after discussion

Authors	Year	Title	Journal	Reason for exclusion
Cristofalo et al	2013	Randomized trial of exclusive human milk versus preterm formula diets in extremely premature infants	Journal of Pediatrics	preterm, HIC
Griffin et al	1999	Can the elimination of lactose from formula improve feeding tolerance in premature infants?	Journal of Pediatrics	preterm, HIC
Lucas et al	1984	Multicentre trial on feeding low birthweight infants: Effects of diet on early growth	Archives of Disease in Childhood	preterm, HIC
Marseglia et al	2015	A new formula for premature infants: effects on growth and nutritional status	Journal of Maternal-Fetal & Neonatal Medicine	preterm, HIC
Mihatsch et al	2002	Hydrolyzed protein accelerates feeding advancement in very low birth weight infants	Pediatrics	preterm, HIC
Schanler et al	2005	Randomized trial of donor human milk versus preterm formula as substitutes for mothers' own milk in the feeding of extremely premature infants	Pediatrics	preterm, HIC
Stefanescu et al	2016	Very low birth weight infant care: Adherence to a new nutrition protocol improves growth outcomes and reduces infectious risk	Early Human Development	preterm, HIC
Toftlund et al	2018	Catch-Up Growth, Rapid Weight Growth, and Continuous Growth from Birth to 6 Years of Age in Very-Preterm-Born Children	Neonatology	preterm, HIC
van Goudoever et al	2000	Short-term growth and substrate use in very-low-birth-weight infants fed formulas with different energy contents	American Journal of Clinical Nutrition	preterm, HIC
Agakidou et al	2018	Modifications of own mothers' milk fortification protocol affect early plasma IGF-I and ghrelin levels in preterm infants. A randomized clinical trial	Nutrients	preterm, HIC
Arslanoglou et al	2006	Adjustable fortification of human milk fed to preterm infants: does it make a difference?	Journal of Perinatology	preterm, HIC
Bhatia et al	1991	Effect of protein-energy ratio on growth and behavior or premature infants	Journal of Pediatrics	preterm, HIC
Biasini et al	2017	High protein intake in human/maternal milk fortification for ≤1250 gr infants: Intrahospital growth and neurodevelopmental outcome at two years	Acta Biomedica	preterm, HIC
Brion et al	2020	Optimizing individual nutrition in preterm very low birth weight infants: double-blinded randomized controlled trial	Journal of Perinatology	preterm, HIC

Casper et al	2016	Recombinant bile salt-stimulated lipase in preterm infant feeding: A randomized phase 3 study	PLoS One	preterm, HIC
Cooke et al	2010	Adiposity Is Not Altered in Preterm Infants Fed With a Nutrient-Enriched Formula After Hospital Discharge	Pediatric Research	preterm, HIC
Ditzenberger et al	2013	Supplemental protein and postnatal growth of very low birth weight infants: a randomized trial	Journal of Neonatal-Perinatal Medicine	preterm, HIC
Dogra et al	2017	Effect of Differential Enteral Protein on Growth and Neurodevelopment in Infants <1500 g: A Randomized Controlled Trial	Journal of Pediatric Gastroenterology and Nutrition	preterm, HIC
Hagelberg et al	1990	Amino-acid levels in the critically ill preterm infant given mothers milk fortified with protein from human or cows milk	Acta Pediatrica Scandanavia	preterm, HIC
Hair et al	2016	Premature infants 750-1,250g birth weight supplemented with a novel human milk-derived cream are discharged sooner	Breastfeeding Medicine	preterm, HIC
Hascoet et al	2022	Use of a Liquid Supplement Containing 2 Human Milk Oligosaccharides: The First Double-Blind, Randomized, Controlled Trial in Pre-term Infants	Frontiers in Pediatrics	preterm, HIC
Khaira et al	2022	Expressed Breast Milk Analysis: Role of Individualized Protein Fortification to Avoid Protein Deficit After Preterm Birth and Improve Infant Outcomes	Frontiers in Pediatrics	preterm, HIC
Lucas et al	2020	Preterm Infants Fed Cow's Milk-Derived Fortifier Had Adverse Outcomes Despite a Base Diet of only Mother's Own Milk	Breastfeeding Medicine	preterm, HIC
Lucas et al	2001	Randomized trial of nutrient-enriched formula versus standard formula for postdischarge preterm infants	Pediatrics	preterm, HIC
Miller et al	2012	Effect of increasing protein content of human milk fortifier on growth in preterm infants born at <31 wk gestation: a randomized controlled trial	American Journal of Clinical Nutrition	preterm, HIC
Rigo et al	2017	Growth and Nutritional Biomarkers of Preterm Infants Fed a New Powdered Human Milk Fortifier: A Randomized Trial	Journal of Pediatric Gastroenterology and Nutrition	preterm, HIC
Salas et al	2022	Body composition of extremely preterm infants fed protein-enriched, fortified milk: a randomized trial	Pediatric Research	preterm, HIC

Wauben	1998	Moderate nutrient supplementation of mother's milk for preterm infants supports adequate bone mass and short-term growth: a randomized, controlled trial	American Journal of Clinical Nutrition	preterm, HIC
Carlson et al	1996	Effect of long-chain n-3 fatty acid supplementation on visual acuity and growth of preterm infants with and without bronchopulmonary dysplasia	American Journal of Clinical Nutrition	preterm, HIC
Kitamura et al	2016	The Ratio of Docosahexaenoic Acid and Arachidonic Acid in Infant Formula Influences the Fatty Acid Composition of the Erythrocyte Membrane in Low-Birth-Weight Infants	Annals of Nutrition & Metabolism	preterm, HIC
Roggero et al	2012	Growth and fat-free mass gain in preterm infants after discharge: A randomized controlled trial	Pediatrics	preterm, HIC
Roggero et al	2011	Small for gestational age preterm infants: Nutritional strategies and quality of growth after discharge	Journal of Maternal-Fetal & Neonatal Medicine	preterm, HIC
Nandakumar et al	2019	Exclusive breast milk vs. hybrid milk feeding for preterm babies - A randomized controlled trial comparing time to full feeds	Journal of Tropical Pediatrics	preterm, LMIC
Nangia et al	2019	Early Total Enteral Feeding versus Conventional Enteral Feeding in Stable Very-Low-Birth-Weight Infants: A Randomised Controlled Trial	Neonatology	preterm, LMIC
Arun et al	2019	An Open-label Randomized Controlled Trial to Compare Weight Gain of Very Low Birth Weight Babies with or without Addition of Coconut Oil to Breast Milk	Journal of Tropical Pediatrics	preterm, LMIC
Chinnappan et al	2021	Fortification of Breast Milk With Preterm Formula Powder vs Human Milk Fortifier in Preterm Neonates: A Randomized Noninferiority Trial	JAMA Pediatrics	preterm, LMIC
Mukhopadhyay et al	2007	Effect of human milk fortification in appropriate for gestation and small for gestation preterm babies: A randomized controlled trial	Indian Pediatrics	preterm, LMIC
Thanh et al	2022	Effects of higher protein formula with improved fat blend on growth, feeding tolerance and nutritional biomarkers in preterm infants: A double-blind, randomized, controlled clinical trial	Pediatrics and Neonatology	preterm, LMIC
Japakasetr et al	2016	Implementation of a nutrition program reduced post-discharge growth restriction in Thai very low birth weight preterm infants	Nutrients	preterm, UMIC
Li et al	2019	Effect of different feeding initiation formulas on very low birth weight infants	Chinese Journal of Contemporary Pediatrics	preterm, UMIC

Yu et al	2020	Effects of a nutrient-dense formula compared with a post-discharge formula on post-discharge growth of preterm very low birth weight infants with extrauterine growth retardation: a multicentre randomised study in China	Journal of Human Nutrition and Dietetics	preterm, UMIC
Yu et al	2014	Effects of extensively hydrolyzed protein formula on feeding and growth in preterm infants: A multicenter controlled clinical study	Chinese Journal of Contemporary Pediatrics	preterm, UMIC
Zuckerman et al	1994	Rickets in very-low-birth-weight infants born at Baragwanath Hospital	South African Medical Journal	preterm, UMIC
Hamidi et al	2022	The Effect of Protein Supplementation on Body Growth Indices and Immune System Development in Premature Neonates with Very Low Birth Weight	Erciyes Medical Journal	preterm, UMIC
Cosgrove et al	1996	Nucleotide supplementation and the growth of term small for gestational age infants	Archives of Disease in Childhood	single nutrient supplementation
Brunton et al	1998	Growth and body composition in infants with bronchopulmonary dysplasia up to 3 months corrected age: a randomized trial of a high-energy nutrient-enriched formula fed after hospital discharge	Journal of Pediatrics	special circumstances, bronchopulmonary dysplasia
Clarke et al	2007	Randomized comparison of a nutrient-dense formula with an energy-supplemented formula for infants with faltering growth	Journal of Human Nutrition and Dietetics	special circumstances, cardiac lesions, cystic fibrosis, other organic causes
DiLauro et al	2020	Growth of cardiac infants with post-surgical chylothorax can be supported using modified fat breast milk with proactive nutrient-enrichment and advancement feeding protocols; an open-label trial	Clinical Nutrition ESPEN	special circumstances, cardiac infants
Dupont et al	2015	An extensively hydrolysed casein-based formula for infants with cows' milk protein allergy: tolerance/hypo-allergenicity and growth catch-up	British Journal of Nutrition	special circumstances, cow's milk protein allergy
Ellis et al	1998	Do infants with cystic fibrosis need a protein hydrolysate formula? A prospective, randomized, comparative study	Journal of Pediatrics	special circumstances, cystic fibrosis
Evans et al	2008	Should high-energy infant formula be given at full strength from its first day of usage?	Journal of Human Nutrition and Dietetics	special circumstances, cardiac infants
Fewtrell et al	1997	Randomized trial of high nutrient density formula versus standard formula in chronic lung disease	Acta Pediatrica, International Journal of Pediatrics	special circumstances, chronic lung disease
Hong-Mei et al	2021	Effect of calorie-enriched formula on postoperative catch-up growth in infants with cyanotic congenital heart disease: a prospective randomized controlled study	Chinese Journal of Contemporary Pediatrics	special circumstances, cyanotic congenital heart disease

Huang et al	2019	Clinical effect of feeding with calorie-enriched formula in children with ventricular septal defect and severe pneumonia	Chinese Journal of Contemporary Pediatrics	special circumstances, ventricular septal defect and pneumonia
McGrath et al	2012	The prevalence of stunting is high in HIV-1-exposed uninfected infants in Kenya	Journal of Nutrition	special circumstances, HIV-exposed
McLeish et al	1995	Comparison of an elemental with a hydrolysed whey formula in intolerance to cows' milk	Archives of Disease in Childhood	special circumstances, cow's milk protein allergy
Pillo-Blocka et al	2004	Rapid advancement to more concentrated formula in infants after surgery for congenital heart disease reduces duration of hospital stay: A randomized clinical trial	Journal of Pediatrics	special circumstances, congenital heart disease
Shulman et al	1989	Human milk and the rate of small intestinal mucosal recovery in protracted diarrhea	Journal of Pediatrics	special circumstances, diarrhea, sterile water as comparison
Velaphi	2008	Growth and metabolism of infants born to women infected with human immunodeficiency virus and fed acidified whey-adapted starter formulas	Nutrition	special circumstances, HIV-exposed
Zhang et al	2019	High-energy nutrition in paediatric cardiac critical care patients: a randomized controlled trial	Nursing in Critical Care	special circumstances, cardiac infants
Fewtrell et al	2001	Catch-up growth in small-for-gestational-age term infants: A randomized trial	American Journal of Clinical Nutrition	wrong intervention
Gathwala et al	2007	Fortified human milk in the small for gestational age neonate	Indian Journal of Pediatrics	wrong intervention, fortifier sachet + EHM
De Zegher et al	2012	Body composition and circulating high-molecular-weight adiponectin and IGF-I in infants born small for gestational age: Breast- versus formula-feeding	Diabetes	wrong outcomes
De Zegher et al	2013	Breast-feeding vs formula-feeding for infants born small-for-gestational-age: divergent effects on fat mass and on circulating IGF-I and high-molecular-weight adiponectin in late infancy	Journal of Clinical Endocrinology and Metabolism	wrong outcomes
Graham et al	1996	Protein requirements of infants and children: Growth during recovery from malnutrition	Pediatrics	wrong population
Alexander et al	2022	Nutritional Management of Moderate- and Late-Preterm Infants Commenced on Intravenous Fluids Pending Mother's Own Milk: Cohort Analysis From the DIAMOND Trial	Frontiers in Pediatrics	wrong study design
Awasthi et al	1989	Is high protein milk beneficial for SGA-terms?	Indian Pediatrics	wrong study design
