# Supplementary material for: Critical research gaps in treating growth faltering in infants under 6 months: A systematic review and meta-analysis
Source: PLOS Glob Public Health. 2024 Jan 8;4(1):e0001860. doi: 10.1371/journal.pgph.0001860 (PMC10773941; doi:10.1371/journal.pgph.0001860)
Supplement: Attachment — Submitted filename: PGPH-D-23-00601_R1+GH.pdf [file pgph.0001860.s007.pdf]

PLOS Global Public Health

Critical Research Gaps in Treating Growth Faltering in Infants under 6 Months: A Systematic Review and Meta-Analysis

--Manuscript Draft--

**Manuscript Number:**	PGPH-D-23-00601R1
**Article Type:**	Research Article
**Full Title:**	Critical Research Gaps in Treating Growth Faltering in Infants under 6 Months: A Systematic Review and Meta-Analysis
**Short Title:**	Critical Research Gaps in Treating Growth Faltering in Infants under 6 Months
**Corresponding Author:**	Cecilia Tomori, PhD, MA<br>Johns Hopkins Bloomberg School of Public Health<br>Baltimore, MD UNITED STATES
**Order of Authors:**	Cecilia Tomori, PhD, MA
	Deborah O'Connor, PhD, RD
	Mija Ververs, MMed, MPH, RD
	Dania Orta-Aleman, PhD, MPH
	Katerina Paone
	Chakra Budhathoki, PhD
	Rafael Pérez-Escamilla, PhD
**Keywords:**	Growth Faltering; malnutrition recovery; wasting; stunting; breastfeeding; relactation; supplemental milk
**Abstract:**	Introduction<br>In 2020, 149.2 million children worldwide under 5 years suffered from stunting, and 45.4 million experienced wasting. Many infants are born already stunted, while others are at high risk for growth faltering early after birth. Growth faltering is linked to transgenerational impacts of poverty and marginalization. Few interventions address growth faltering in infants under 6 months, despite a likely increasing prevalence due to the negative global economic impacts of the COVID-19 pandemic. Breastfeeding is a critical intervention to alleviate malnutrition and improve child health outcomes, but rarely receives adequate attention in growth faltering interventions.<br>Methods<br>A systematic review and meta-analysis were undertaken to identify and evaluate interventions addressing growth faltering among infants under 6 months that employed supplemental milks. The review was carried out following guidelines from the USA National Academy of Medicine.<br>Results<br>A total of 10,405 references were identified, and after deduplication 7390 studies were screened for eligibility. Of these, 227 were assessed for full text eligibility and relevance. Two randomized controlled trials were ultimately included, which differed in inclusion criteria and methodology and had few shared outcomes. Both studies had small sample sizes, high attrition and high risk of bias. A Bangladeshi study (n=153) found significantly higher rates of weight gain for F-100 and diluted F-100 (DF-100) compared with infant formula (IF), while a DRC trial (n=146) did not find statistically significant differences in rate of weight gain for DF-100 compared with IF offered in the context of broader lactation and relactation support. The meta-analysis of rate of weight gain showed no statistical difference and some evidence of moderate heterogeneity.<br>Conclusion<br>Few interventions address growth faltering among infants under 6 months. These studies have limited generalizability and have not comprehensively supported lactation. Greater investment is necessary to accelerate research that addresses growth faltering following a new research framework that calls for comprehensive lactation support.
**Additional Information:**	

Powered by Editorial Manager® and ProduXion Manager® from Aries Systems Corporation

Question	Response
**Financial Disclosure**<br><br>Enter a financial disclosure statement that describes the sources of funding for the work included in this submission and the role the funder(s) played. This includes grants and any commercial funding of the work or authors.<br><br>This statement will be typeset if the manuscript is accepted for publication.<br><br>*Please review the submission guidelines and the instructions link below for detailed requirements and guidance.*	This study was funded by the World Health Organization (WHO). WHO commissioned the review and as such provided guidance on the development of the questions examined by the review, and reviewed and provided methodological feedback on the technical report. WHO did not review or provided feedback on the interpretation of findings or recommendations from this article. These were made by the article's authors and do not necessarily represent the views from the WHO.
**Competing Interests**<br><br>On behalf of all authors, disclose any competing interests that could be perceived to bias this work.<br><br>This statement will be typeset if the manuscript is accepted for publication.<br><br>*Please review the instructions link below and PLOS Global Public Health's competing interests policy to determine what information must be disclosed at submission.*	The authors declare no competing interests.
**Data Availability**<br><br>Before publication, Authors are required to make fully available and without restriction all data underlying their findings. Please see our PLOS Data Policy page for detailed information on this policy.<br><br>A **Data Availability Statement**, detailing where the data can be accessed, is	Data for this systematic review are presented in the manuscript and supporting documentation.

Powered by Editorial Manager® and ProduXion Manager® from Aries Systems Corporation

required at first submission. Insert your Data Availability Statement in the box below.<br><br>Please see the data reporting section of our submission guidelines for instructions on what you need to include in your Data Availability Statement.<br><br>This statement will be typeset if the manuscript is accepted for publication.<br><br>*PLOS allows rare exemptions to address legal and ethical concerns. If you have legal or ethical restrictions, please use the box below to detail these in full sentences for the Journal team to consider.*	

Manuscript

Click here to access/download;Manuscript;Growth faltering revised 8-10-23 clean.docx

Critical Research Gaps in Treating Growth Faltering in Infants under 6 Months: A Systematic Review and Meta-Analysis

Cecília Tomori, PhD[1, 2]; Deborah L O'Connor, PhD, RD[3]; Mija Ververs, MMed, MPH, RD[2]; Dania Orta-Aleman, MPH[2]; Katerina Paone[4]; Chakra Budhathoki[1], PhD; Rafael Pérez-Escamilla, PhD[4]

[1] Johns Hopkins University School of Nursing, Baltimore, MD, USA
[2] Johns Hopkins University Bloomberg School of Public Health, Baltimore, MD, USA
[3] University of Toronto, Department of Nutritional Sciences, Temerty Faculty of Medicine, Toronto, Ontario, Canada
[4] Yale University School of Public Health, New Haven, CT, USA

Abstract

Introduction

In 2020, 149.2 million children worldwide under 5 years suffered from stunting, and 45.4 million experienced wasting. Many infants are born already stunted, while others are at high risk for growth faltering early after birth. Growth faltering is linked to transgenerational impacts of poverty and marginalization. Few interventions address growth faltering in infants under 6 months, despite a likely increasing prevalence due to the negative global economic impacts of the COVID-19 pandemic. Breastfeeding is a critical intervention to alleviate malnutrition and improve child health outcomes, but rarely receives adequate attention in growth faltering interventions.

Methods

A systematic review and meta-analysis were undertaken to identify and evaluate interventions addressing growth faltering among infants under 6 months that employed supplemental milks. The review was carried out following guidelines from the USA National Academy of Medicine.

Results

A total of 10,405 references were identified, and after deduplication 7390 studies were screened for eligibility. Of these, 227 were assessed for full text eligibility and relevance. Two randomized controlled trials were ultimately included, which differed in inclusion criteria and methodology and had few shared outcomes. Both studies had small sample sizes, high attrition and high risk of bias. A Bangladeshi study (n=153) found significantly higher rates of weight gain for F-100 and diluted F-100 (DF-100) compared with infant formula (IF), while a DRC trial (n=146) did not find statistically significant differences in rate of weight gain for DF-100 compared with IF offered in the context of broader lactation and relactation support. The meta-analysis of rate of weight gain showed no statistical difference and some evidence of moderate heterogeneity.

Conclusion

Few interventions address growth faltering among infants under 6 months. These studies have limited generalizability and have not comprehensively supported lactation. Greater investment is

necessary to accelerate research that addresses growth faltering following a new research framework that calls for comprehensive lactation support.

Introduction

A substantial portion of infants face growth faltering each year across the globe[1-4]. While definitions vary, for the purpose of this paper the term growth faltering refers to a slower rate of weight, length or head circumference gain than expected for a children's age and sex [5]. Of the 149 million children globally under 5 years who are stunted (length-for-age z score less than 2 standard deviation scores below the World Health Organization (WHO) child growth standards median[6]), recent research indicates that 15% are born stunted, and an additional 25% become stunted during the first 6 months after birth[7]. Additionally, 45.4 million children under 5 years of age, constituting approximately 6.7% of children, experienced wasting (weight-for-length z score less than 2 standard deviation scores below the WHO child growth standards median[6])[4]. Infants who experience growth faltering and do not receive adequate nutritional interventions are at increased risk for mortality and morbidity from infectious illness and from non-communicable diseases[8-11]. The causes of growth faltering are complex and multifactorial. Preterm births (PTB), and infants born with low birth weight (LBW, <2500g), and small for gestational age (SGA, birth weight <10th percentile) constitute a substantial portion of those at risk across the globe for growth faltering[6,12]. Multiple comorbidities may increase the risk of growth faltering.

Importantly, social determinants of health (SDOH), such as poverty and marginalization, are key drivers of conditions that put infants at risk for growth faltering. For instance, mothers with short stature experience higher rates of LBW and preterm births[13]. Short stature itself is the outcome of intergenerational poverty[14]. Impoverished women are at higher risk for infectious diseases such as malaria and HIV, both causes of PTB, LBW and SGA[15-17]. COVID-19 is also associated with a higher risk of preterm birth[18] – and with inequities. Poverty also drives exposures to infectious agents in the home environment due to lack of access to proper sanitation including clean water, toilets, clean housing among other factors.[19,20] Poor and marginalized women across settings are at high risk for a number of complications during pregnancy[21] including diabetes and hypertension, which are associated with a substantial burden of adverse birth outcomes[22,23]. Importantly, as the food environment is rapidly changing in many settings, people in LMIC are often facing a double burden of malnutrition, with rising childhood obesity and increasing stunting at the same time. [24,25]Additional environmental drivers of PTB, LBW and SGA[26,27] include indoor and outdoor air pollution, which again disproportionately affects those in poverty. High environmental temperatures are also associated with growth faltering, which is becoming a pervasive threat with the growing impacts of the climate crisis with similarly disproportionate impacts[28].

Maternal mental health also plays a substantial role[29-31] in growth faltering and is itself linked to poverty and related biological and psycho-emotional life stressors including household food insecurity. Genetic anomalies and developmental conditions, such as cleft palate[32], and heart conditions[33] are also drivers of growth faltering. Some of these conditions then also lead to maternal poor mental health, so this relationship can be circular[30]. Conversely, social support is a key mitigator of adverse mental health outcomes,[30] demonstrating the importance of social dynamics in caring for faltering infants. Beyond the other social determinants of adverse birth outcomes mentioned above, additional social drivers of growth may also entail broader gender power dynamics, with women's empowerment and agency linked to infant and child growth.[34,35]

Previous work has identified substantial gaps in the availability of interventions that effectively address growth faltering during infancy and early childhood. A key element that such interventions would need to consider is whether mothers have an enabling breastfeeding environment in which skin to skin contact, early initiation of and exclusive breastfeeding are supported[2,36]. Moreover, when mothers breastfeeding confidence is undermined and there is a lack of breastfeeding support, they are more likely to introduce supplements or end breastfeeding prematurely, increasing risk of infection[37,38], and hence growth faltering. Furthermore, even when supplementation with commercial milk formula (CMF) and/or specially formulated formulas and foods have the potential for increasing growth, there are also potential risks both from the greater velocity of catch up growth and corresponding metabolic consequences as well as from greater risk of infection [39].

Specific evidence on elements of successful growth interventions is often lacking. In large cross-sectional survey in Africa, positive maternal education (years of schooling) and prenatal visits were associated with improved linear growth even in conditions of poverty, highlighting protective factors that could be the basis of future interventions [40]. Critically, kangaroo mother care (KMC) and best breastfeeding practices are rarely fully addressed in available studies[24,36,41,42]. A recent scientific consultation addressing growth faltering in India highlighted some of these research gaps as well as the inadequate follow-up after transitioning from facility-based services to the home-setting[43]. Further strategies that are now standard of care in clinical settings in many middle- and high-income countries to preserve human milk feeding in very preterm and medically compromised infants such as increasing feeding volumes, nutrient-enriching expressed human milk with commercial fortifiers or less costing nutrient modulars (e.g. protein, calcium, phosphorus) or use of donor milk have received little consideration in the context of the late-preterm or term-born infant with growth faltering[41,42]. Additionally, feeding interventions may not be adequate on their own to address growth faltering since some of the drivers require other medical treatment and/or addressing broader environmental factors.

This systematic review was commissioned by the World Health Organization to address questions about optimal supplementation of infants under 6 months of age experiencing growth faltering to inform discussion of the guidelines on treatment of infants and young children with wasting and/or oedema. The review did not examine nutritional interventions aimed at breastfeeding mothers of growth faltering infants. The framework for the review and related recommendations are to be understood in the context of best practices for infant feeding and care, which entail appropriate protection, promotion and support for breastfeeding.

Methods

This study was a systematic review and meta-analysis following the US National Academies of Sciences, Engineering, and Medicine (NASEM) guidelines. The protocol was registered with PROSPERO (CRD42022350150). The review questions were:

1. In infants under 6 months with growth faltering, which criteria best determine if and when an infant should be given a supplemental milk (in addition to breastmilk if the infant is breastfed)?
2. In infants under 6 months with growth faltering meeting criteria in question 1, what is the most effective supplemental milk and for how long should it be given?

Supplemental milk was defined as donor human milk, fortified donor human milk, fortified expressed mothers' own milk, human milk from wet nurse, commercial infant formula, nutrient enriched commercial infant formula, F-75, F-100, or diluted F-100.

Searches were conducted to examine interventions addressing infants under 6 months with growth faltering that used supplemental milks as part of their treatment. Searches were framed as broadly as possible to capture any potentially relevant literature related to the initial questions. Primary outcomes of interest were anthropometry, mortality, clinical deterioration, morbidity, relapse, readmission, and non-response to treatment. Randomized controlled trials, cluster-randomized trials, and quasi-experimental studies/non-randomized controlled studies were included. Qualitative studies, reviews, and systematic reviews, as well as observational studies were excluded. All countries and settings were included.

Searches

The following databases was searched electronically in August 2022: MEDLINE, Embase, CINAHL, Web of Science, Cochrane Library, Global Index Medicus, State of Acute Malnutrition, Emergency Nutrition Network (Field Exchange), eLENA (WHO), UNICEF, FAO, WFP, FAQR, and ClinicalTrials.gov, ISRCTN registry, ICMJE registry, World Health Organization (WHO) International Clinical Trials Registry Platform. The initial searches were designed, tested, and run by a well-trained team that includes public health scientists with expertise in breastfeeding research and a medical librarian. The search strategy developed during preliminary searches are presented in Appendix 1. Search results were combined and de-duplicated in EndNote by the JHU Welch Library Team. Experts in the field were consulted to identify additional articles to be included.

Data Extraction

The study was carried out following the guidance of Preferred Reporting Items for Systematic Reviews and Meta-Analyses (PRISMA)[44]. Search results were exported into EndNote, de-duplicated, and uploaded into Covidence systematic review software for screening. Two research assistants independently screened for potential inclusion of all titles and abstracts identified through the search. Any discrepancies were resolved by consensus or contacting a third author. Attempts were made to contact authors of included studies to obtain clarifications or additional data. Following title and abstract screening, full texts were independently screened for inclusion. Any disagreements were resolved through discussion or by consulting a third review author, if required. Reasons of exclusion were recorded for all the studies excluded at the stage of full text screening. The results of the search are presented using the PRISMA flow diagram.

Structured forms were used for data extraction of key variables including study characteristics and outcomes in a standardized data collection form. Two review authors independently extracted data and discrepancies were resolved through discussion until consensus had been achieved or by consulting a third reviewer, if required.

Risk of bias (quality) assessment

The updated Cochrane risks of bias tool, ROB-2, was used for randomized trials[45]. Two independent authors assessed quality of all eligible studies and disagreements were resolved by consensus or contacting a third author. For all data extraction and study quality assessments two researchers were standardized against each other with screening, data extraction and to assess the quality of each study.

Data synthesis

Statistical heterogeneity was assessed using τ^2 (among study variance), I^2 (degree of inconsistency), and significance of the χ^2 (chi-square) test; and visual inspection of forest plots. Based on prior clinical knowledge, we expected clinical and methodological heterogeneity in included studies and therefore, attempted to explain any observed statistical heterogeneity. The Grading of Recommendations, Assessment, Development and Evaluations (GRADE)[46] methodology was used to assess the certainty of the evidence by outcome. Two independent authors assessed quality of all eligible studies and disagreements were resolved by consensus or contacting a third author. Evidence profiles were generated via GRADE Pro Software.

Results

A total of 10405 references were identified by the searches and imported for screening (Figure 1). Of these, 3015 duplicates were removed. 7390 studies were screened against title and abstract, 7163 studies were excluded, and 227 studies were assessed for full text eligibility. A total of 158 were excluded because the studies were not fully reported (n=50 study protocols or conference abstracts), had the wrong patient population (n=30), the wrong study design (n=26), the wrong intervention (n=20), the wrong outcomes (n=14), or were duplicates (n=4). Full texts were unavailable for 10 studies. Additionally, 4 were carried out prior to 1980 after which significant changes took place in the composition of infant formulas, yielding 69 studies that were published between 1980 and August 2022, when the searches were carried out. [Figure 1 here]

Of the remaining 69 studies, 60 were excluded because they focused on: 1) preterm, predominately hospitalized infants primarily from high income countries 2) infants who received single nutrient supplementation, 3) infants with other special circumstances (e.g., recovering from cardiac surgery) (Appendix 2). Two studies within the infants with special circumstances category were examined further for relevant contextual information (Clarke et al[47] and Evans et al [48]) but were ultimately excluded because the study sample was principally comprised of infants with a significant medical condition known to elevate nutritional requirements and impact growth. Only one infant (of 30) received breastmilk in a previous feed in Evans et al, while Clarke did not report any breastfeeding or receipt of breast milk, making it likely participant infants were nearly all exclusively formula-fed in both studies during the treatment period.

Evans et al[48] enrolled 30 growth faltering infants with a median age of 14.5 weeks (range 2-43 weeks) who were receiving care at a large British specialty referral hospital, 73% (n=22) of whom had cardiac defects. They compared a ready to feed high-energy (1kcal/ml) nutrient-dense (concentrated macro- and micronutrients) infant formula at full strength (25 mOsm kg)1 H_2O) from day one with stepwise introduction of the same ready to feed infant formula but in a diluted form and graded to full strength over three days; infants were studied for a duration of 2 weeks. While

younger infants in the high energy nutrient dense-formula group had an initial increased number of stools, no differences in feeding tolerance nor growth were observed between treatment arms.

Clarke et al[47] enrolled 60 growth faltering infants with a median age of 5 weeks (range 2-31) with cardiac lesions, cystic fibrosis, or other organic causes from Birmingham Children's Hospital in the UK. This open RCT compared two specialty infant formulas, 'high-energy nutrient-dense formula' and a 'high energy only-supplemented formula,' for a period of 6 weeks. The energy concentration of both formulas was 1 kcal/ml; energy was supplied in the "high energy only-supplemented formula" by adding glucose whereas in the "high energy nutrient-dense formula" it was supplied by protein and glucose. The protein concentration of the high energy only and high energy nutrient dense formulas was 1.4 and 2.6 grams/100 kcal, respectively. Infants in the "high energy nutrient-dense formula" group had higher intakes of protein, vitamins and minerals and blood urea nitrogen levels suggesting more favorable protein balance; further there was less fall off of length for age-z-scores compared to the "high energy only-supplemented" group.

An additional seven studies were determined to have the wrong intervention, outcomes, population, or study design (Appendix 2). While initial searches undertaken were purposefully broad, in the full text review of manuscripts it became clear that most included studies focused on preterm babies in high-income settings and/or addressed special populations with particular medical conditions. These studies did not address treatment and recovery from malnutrition, which was the purpose of this review. Therefore, after careful consideration 67 additional studies were excluded, yielding two final studies for consideration.

Two studies ultimately generated direct evidence about the review questions (Islam et al [49] and Wilkinson [50]). Islam et al[49] was published in the peer reviewed literature, while Wilkinson[50] was initially identified in the grey literature, and the full report was provided by the author to the review team.

Included studies

Islam et al

Islam et al was a three-armed randomized controlled trial carried out in Bangladesh in 2012-2015 comparing F-100 (1 kcal/ml, 2.9 g protein/ 100ml; n=50), diluted F-100 (0.77 kcal/ml, 2.2 g protein/100 ml; DF-100, n=52) and standard infant formula (0.70 kcal/ml, 1.6 g protein/100 ml; IF, n=50) in the context of infant rehabilitation from severe acute malnutrition (SAM) (Table 1 below). Islam et. al included infants under 6 months of age admitted for diarrheal illness with weight-for-length z-score of <-3 and/or bipedal edema that were stabilized in a ward for infants requiring long term specialized care less than nine days (free of edema, with cessation of vomiting, fewer than three watery stools /day and no clinical signs of infection). Infants in the three study arms were comparable in most biomedical, socio-economic and demographic characteristics, however, those in the F-100 group were significantly more wasted at the time of enrollment in the trial compared to infants in the other two study arms (p=0.02).

Once stabilized per the above criteria, infants were provided with F-100, DF-100 or standard IF. During the first two days infants received the study diet of 130 ml/kg/day in 12 feeds and then increased by 10 ml per feed until some remained. They were fed every three hours thereafter. Infants were discharged when they gained 15% of their weight or had edema-free weight-for-length z-score ≥ -2. Mothers who were breastfeeding their participant infants were encouraged

to continue doing so. Notably, only approximately half of the participants were breastfed on entry into the trial. Additionally, those who were breastfed received a very small proportion of their feeds from breastfeeding (e.g., less than 10% of total kcal/kg/day, on average). Water was also offered between feeds.

If infants became ill during the treatment, they were returned to the ward for infants requiring long term specialized care or another ward for short-term specialized care, fed F-75 (0.75 kcal/ml and 0.9 g protein/100 ml), and returned to the trial once they recovered from illness. A third of participants (n=52; F-100 n=19; DF-100 n= 20; IF n=13) experienced illness (diarrhea and pneumonia) that necessitated transfer to one of the longer term or specialty wards. The number of infants transferred did not statistically differ across the three arms (p=0.29). No information was provided about the length of treatment in the two other wards or additional details of comparability upon returning to the trial.

Wilkinson

Wilkinson's study was a two-armed study comparing DF-100 (0.73 kcal/ml, 1.8 g protein/100 ml, n=72) and a standard infant formula (0.77 kcal/ml, 1.95 g/100 ml; IF, n=74) in the context of a Management of Acute Malnutrition in Infants (MAMI) project (Table 1 below). This study was designed to improve care for acutely malnourished infants under 6 months in Niger and two sites in the Democratic Republic of Congo (DRC) in 2007-2008. It is important to note that data from Niger were ultimately not presented in the final analysis due to small sample size and lack of adherence to the protocol. Due to fewer than expected visits to Therapeutic Feeding Centers (TFCs) during the study period, the study was not adequately powered to detect differences in treatment.

Wilkinson included infants under six months free of any oedema, with maternal report of breastfeeding failure or not gaining weight at home. No blinding was possible due to the appearance and preparation of the supplemental milks. All mothers of participants were breastfeeding or were engaging in relactation and received lactation support; though this was not quantitated in the report. They used the supplementary suckling technique (SST) to stimulate relactation while receiving therapeutic feeding whenever possible. Supplementary feeds were reduced by half when infants were gaining 20 g/day. Finally, supplementation was stopped when infants maintained a weight gain of 10g/day for 3 days in a row. Infants were discharged when they were able to maintain weight gain for 3-5 days. If infants became ill, they were transferred to a hospital and continued to receive supplemental milk. Breastfeeding observations were not continued during hospitalization due to lack of staff. The author's description indicates that the ill infants who were transferred to the hospital remained in the trial although many of whom later died; it is not clear, however, how well protocols were followed for this group. The author notes that pediatric and neonatal services were limited and weak at the hospitals to which ill infants were transferred, with no specialized neonatal care for very young infants.

Attrition, final sample size, and risk of bias

Islam et al experienced high attrition of 24 infants (16%) leaving the trial before discharge criteria were met across all three arms. Attrition did not statistically differ across the three study arms. Supplementary data suggest that 8 additional infants dropped out of the study (see Islam et al Supplementary Table 2). One infant in the F-100 died due to a hospital acquired infection.

Consequently, only 36/52 (F-100), 42/52 (DF-100), 42/50 (IF) infants from the three groups, respectively, completed the study to discharge. Islam et al was rated at high risk of bias according to the ROB-2 assessment due to non-adherence to the assigned intervention and loss to follow-up (see Table 1 and Tables 2-4 for details).

The Wilkinson trial also experienced high rates of infants leaving the trial prior to meeting discharge criteria (DF-100 n=13; IF n=10) (no difference between study arms). Additionally, there was a large number of deaths in both arms (DF-100 n=12; IF n=11) (no difference between study arms). One infant from each group was transferred out of the study. A total of 48 infants were recovered in the DF-100 and 50 in the IF group, respectively. These findings did not differ by intervention. Wilkinson's study was rated at high risk of bias according to the ROB-2 assessment due to unblinding and loss to follow-up (see Table 1 and Tables 2-4 for details).

Outcomes of interest

Due to the small number of studies identified by the search, the initial questions for the review could not be fully assessed. We present the outcomes of interest that are available in three GRADE tables (Tables 2-4 below). Table 2 combines the comparison shared by the two included studies (DF-100 compared with IF). Detailed information about the populations and interventions is presented in Table 2 footnotes a) and b) to help contextualize the findings. Additionally, Table 3 presents the F-100 v. IF comparison, and Table 4 presents the F-100 v DF-100 comparison, both from Islam et al.

Rate of weight gain was the only outcome assessed for DF-100 v IF in both studies. Islam et al found significantly higher rates of weight gain for both F-100 (mean difference 4.6; 95% CI 1.5-7.6; p=0.004) and DF-100 (mean difference 3.1; 95% CI 0.6-5.5; p= 0.015) when compared with IF, respectively. In contrast, Wilkinson did not find significant differences in rate of weight gain between DF-100 and IF (0.1 g/kg/day greater for IF-100, 95% CI: -3.3, 3.5).

The weight gain outcome (g/kg/day) was meta-analyzed with the two studies (Islam et al 2019 and Wilkinson 2008) using a random-effects model (Figure 2). The meta-analysis estimate of the mean difference in weight gain between IF and DF-100 was 1.72 g/kg/day, which was not statistically significant (p=0.278); 95% CI: -1.39, 4.83. There was some evidence of moderate heterogeneity across studies, although it was not statistically significant; Q(1)=2.30, p=0.130; I^2=56.5.

Total weight gain at discharge was only reported for Wilkinson and did not significantly differ between the two groups (non-significantly higher weight gain for DF-100 compared with IF; 63.8 g (95% CI: -53.4, 181).

There was one death in Islam's study due to a hospital acquired infection, but it was deemed unrelated to the infant's diet. Mortality was high in both arms of the Wilkinson study (DF-100 n=12; IF n=11) but did not differ by treatment arm. Infants who died were all under 60 days old at admission; 43% (10/23) were less than 30 days old at admission, and 56% (13/23) were less than 60 days old at admission.

Islam et al reported faster recovery for the F-100 group than for the IF group (p=0.043), but the rate of recovery for the F-100 group was not faster than for infants who received DF-100 (p=0.09). There were no differences in rate of recovery or duration of treatment from admission to discharge between the two arms of the Wilkinson study overall. However, there were significant differences between the two centers in duration of treatment, with significantly shorter treatment from admission to discharge for IF compared with DF-100 in Baraka (4.7 days 95% CI: -10.7, 1.3) and significantly longer treatment from admission to discharge for the IF arm compared with DF-100 in Uvira (3.5 days 95% CI: -1.5, 8.5).

Islam et al also examined additional outcomes to assess the safety of F-100 based on previous concerns that it would result in high renal solute load (RSL) and risk of hyponatraemic dehydration. The study did not find evidence of these potential safety issues. The RSL was highest in the F-100 group (126.4 51.9 ± 51.9 mOsm/day) and was significantly different between F-100 and DF-100 as well as between F-100 and IF groups. However, sodium and urinary specific gravity were within normal range (Tables 2-4).

Discussion

The Islam et al trial recommended the use of undiluted F-100 as a simpler approach and did not find any safety concerns in the controlled, hospital environment of the trial. Although it should be noted that none of the infants in the trial had diarrhea, nor had dehydration. This is because in that trial infants who became ill with diarrhea, were temporarily transferred out of the trial and received F-75 coupled with additional treatment. In field practice, however, many growth faltering infants have diarrhea, may be febrile, and/or are fluid restricted for a variety of reasons (e.g. those with cardiac defects, those who are mixed fed and lack access to clean drinking water, etc.). In these situations, the higher solute load of F-100 may pose a risk.

Wilkinson's approach emphasized the use of the supplemental suckling technique (SST) to facilitate relactation and lowered the amount of supplemental milk as infants recovered. However, the trial did not have sufficient resources to record further breastfeeding data or to support those who were transferred to under-resourced hospitals that lacked specialized pediatrics care and skilled lactation support. The study population of Islam et al reflects a very short period of exclusive breastfeeding and any breastfeeding at the time of trial (~50%). Although mothers who were breastfeeding received encouragement to continue and those non-breastfeeding were counseled on relactation, infants only received a very small portion of their energy intake from breastfeeding. Additionally, infants were offered water between feeds, which is not consistent with WHO guidance.

The trials in this review demonstrate the continued need to support lactation in the context of growth faltering as part of an integrated approach protecting, promoting, and supporting breastfeeding. Although WHO guidance is clear that exclusive breastfeeding for 6 months ensures optimal infant feeding and development, to date, the focus of growth faltering interventions has been on the supplemental milks that may be used in these contexts without paying enough attention to supporting breastfeeding or relactation. A recent observational study[51] of LBW infants in resource limited settings similarly identified a need for "universal and consistent" lactation support. Moreover, a recent systematic review[52] identified additional need for high quality studies of interventions to address breastfeeding difficulties among mothers of

infants under 6 months with growth faltering. Our review identified a study protocol that addresses growth faltering in infants under 6 months in Asia and Africa in the context of fully implementing WHO Breastfeeding Counselling Guidelines on breastfeeding promotion and support (PI: Bhandari).[53] This study has not started recruitment.

Overall, there is very limited high-quality evidence on interventions to understand the nutritional management of infants under 6 months experiencing growth faltering. Of the two trials identified by this search, only one has been published in the peer reviewed literature. Both trials had small sample sizes, and high numbers of participants leaving the trial before discharge criteria were met. This limits the generalizability of the findings. In Islam's trial the authors noted that the F-100 study arm differed between the other two study arms in greater wasting at enrollment. Additionally, over a third of infants temporarily paused their participation in the trial while transferred out due to illness, and then were later returned to the trial, which poses methodological challenges for comparability. For Wilkinson's trial the inability to adequately power the trial and the lack of blinding posed challenges. There were additional non-statistically significant differences in the findings between the two centers, with longer duration of treatment from admission to discharge for the diluted F-100 group at Baraka versus longer duration for the IF group at Uvira. Finally, the design of the two studies differed substantially, and only one outcome (rate of weight gain) was shared among them for the diluted F-100 and IF comparison. The lack of studies combined with the low quality of the only two studies available is quite concerning given the strong need for evidence-based guidelines for the nutritional management of young malnourished children. This gap has been identified for decades. It is indeed striking that many national guidelines on the treatment of acutely malnourished children include F-75, diluted F-100 or infant formula for infants under 6 months in the recommendations without having the level of evidence needed to support this.

Another key concern in this area is the lack of a well-thought-out framework that can help improve the design for studies in this area, which accounts for the need to first and foremost protect breastfeeding among malnourished infants. A current Cochrane review is examining whether lower protein content formulas can address malnutrition without the adverse longer-term outcomes of obesity and metabolic disorders[54]. High income settings face additional considerations on the use of fortifiers and modular nutrients for preterm infants, including cost and cross-contamination of using pumps to express milk to nutrient enrich a supply.[55]

We specifically propose a new research framework that is consistent with the MAMI pathway[56] package's emphasis on continuing/restoring breastfeeding whenever possible, and supports continuation of breastfeeding, relactation counseling, and expands this framework with the use of human milk. We recommend conducting a series of linked studies to inform an evidence-based decision tree to understand if, when and for how long the malnourished child needs to be supplemented with supplemental milks and which kind of supplemental milks are needed (Figure 3). In full consistency with our systematic review's conclusion, the revised 2023 WHO guideline on the prevention and management of wasting and nutritional oedema (acute malnutrition) in infants and children under 5 years also emphasizes the importance of supporting and restoring optimal breastfeeding practices to prevent malnutrition and in the context of continued support for recovery from acute malnutrition[57]. In caring for hospitalized infants that are at elevated risk of growth faltering (e.g. very low birth weight infant or infants with cardiac defects), there has been a paradigm shift in the past 30 years from the almost exclusive use of nutrient-enriched specialty formulas to a variety of strategies to preserve human milk feeding,

but without rigorously designed research studies. These strategies have included breastfeeding support, increasing the volumes of breast milk fed, and adding nutrient fortifiers or less expensive nutrient modulars to expressed human milk (mother's own or donor human milk) fed by feeding bottle, tube or a supplemental nursing system. Recent WHO guidance on caring for preterm and LBW infants states that mother's own milk (MOM) is recommended for this group, and there is limited to no benefits to using IF in comparison to MOM[6]. The evidence base for the guidance suggests that breastfeeding is likely an undervalued intervention for these vulnerable infants. Although this guidance is not specifically directed at infants with growth faltering, it has implications for interventions to address growth faltering in settings with high prevalence of malnutrition. Given that practitioners need to make feeding decisions with very little evidence to guide them, it is important to also learn from successful practical experiences managing the nutritional needs of infants who are not growing well across diverse settings, and carefully evaluate them.

The trials also call attention to the importance of translation of research through implementation of interventions that is responsive to the needs of diverse local contexts with a high prevalence of growth faltering. A key issue for both trials in our analysis was that there were infants leaving the trial before discharge criteria were met. When caregivers have competing demands on their time, they may struggle to keep their infants in treatment, and sustaining recovery may also be a challenge. They may have labor and other caregiving demands that prevent them from being able to stay at a hospital or recovery center. To ensure recovery and sustained growth and healthy development, a broader evidence-informed policy approach is necessary to ensure support for families facing these challenges.

Conclusion and research recommendations

Critical questions remain about how to best prevent, and support recovery, from growth faltering in the context of malnutrition. The two studies we identified in this systematic review provide limited generalizability. The inequities in the prevalence of growth faltering under 6 months between those in low- and middle-income settings compared with high-income settings point to the importance of addressing the broader social inequities that are the upstream drivers of malnutrition.[58] Additional research needs to address the needs of infants at risk for growth faltering and those facing complex medical conditions. We recommend a paradigm shift for this research that reorients breastfeeding and the use of human milk and skin to skin contact as foundational even to medically fragile infants. In this paradigm, the focus in malnutrition research would be on supporting and restoring breastfeeding, rather than previous approaches that focus research questions on supplemental milk products. A similar paradigm shift has already taken place in preterm infant feeding research and practice, as reflected by recent WHO guidance[6]. Further investment is necessary to ensure that the protection, promotion and support of breastfeeding is at the center of this work, even in complex humanitarian emergencies.

Acknowledgements
The authors would like to thank the Johns Hopkins University Welch Library team for their assistance.

Data availability statement

All data are publicly available. The search strategy as well as the included and excluded studies can be found in the manuscript.

Author contributions

CT, DOC, MV, and RPE conceptualized the study. DOA and KP carried out the review and analyses of findings, supported by the entire author team. CB provided additional analyses. All authors contributed to the development of the final manuscript.

Competing interests statement

The authors declare no competing interests.

Funding

This study was funded by the World Health Organization (WHO). WHO commissioned the review and as such provided guidance on the development of the questions examined by the review, and reviewed and provided methodological feedback on the technical report. WHO did not review or provided feedback on the interpretation of findings or recommendations from this article. These were made by the article's authors and do not necessarily represent the views from the WHO.

Captions

Figure 1. PRISMA flow chart

Figure 2. Meta-analysis of weight gain

Figure 3. Research framework to guide the design of future studies to inform comprehensive evidence-based guidelines for recovery from growth faltering in infants <6 months. Note: Supplemental milk is defined as donor human milk, fortified donor human milk, fortified mothers' own milk, human milk from wet nurse, commercial infant formula, nutrient-enriched commercial infant formula, F-75, F-100, or diluted F-100.

Table 1. Summary of included studies (Islam et al 2019, Wilkinson 2008).

Table 2. GRADE Evidence Profile: Diluted F-100 compared to infant formula for infants <6 months of age with severe wasting and/or oedema Author(s): Tomori et al.
Question: Diluted F-100 compared to infant formula for infants <6 months with severe wasting and/or oedema.

Table 3. GRADE Evidence Profile: F-100 compared to infant formula for infants <6 months of age with severe wasting and/or oedema Author(s): Tomori et al.
Question: F-100 compared to infant formula for infants <6 months with severe wasting and/or oedema.

Table 4. GRADE Evidence Profile: F-100 compared to diluted F-100 for infants <6 months of age with severe wasting and/or oedema Author(s): Tomori et al.

Question: F-100 compared to diluted F-100 for infants <6 months with severe wasting and/or oedema

References

1. Victora CG, De Onis M, Hallal PC, Blössner M, Shrimpton R. Worldwide timing of growth faltering: revisiting implications for interventions. *Pediatrics* 2010; **125**(3): e473-e80.
2. Rana R, McGrath M, Gupta P, Thakur E, Kerac M. Feeding Interventions for Infants with Growth Failure in the First Six Months of Life: A Systematic Review. *Nutrients* 2020; **12**(7): 2044.
3. Kerac M, Blencowe H, Grijalva-Eternod C, et al. Prevalence of wasting among under 6-month-old infants in developing countries and implications of new case definitions using WHO growth standards: a secondary data analysis. *Archives of disease in childhood* 2011; **96**(11): 1008-13.
4. UNICEF/WHO/World Bank Group. Levels and trends in child malnutrition. Geneva, Switzerland: UNICEF, WHO, World Bank Group, 2021.
5. National Institute for Health Care Excellence. Faltering growth: recognition and management of faltering growth in children. NICE London (United Kingdom); 2017.
6. World Health Organization. WHO recommendations for care of the preterm or low-birth-weight infant. Geneva, Switzerland: World Health Organization, 2022.
7. Benjamin-Chung J, Mertens A, Colford JM, et al. Early childhood linear growth faltering in low- and middle-income countries. *medRxiv* 2021: 2020.06.09.20127001.
8. Lelijveld N, Seal A, Wells JC, et al. Chronic disease outcomes after severe acute malnutrition in Malawian children (ChroSAM): a cohort study. *The Lancet Global Health* 2016; **4**(9): e654-e62.
9. Grey K, Gonzales GB, Abera M, et al. Severe malnutrition or famine exposure in childhood and cardiometabolic non-communicable disease later in life: a systematic review. *BMJ global health* 2021; **6**(3): e003161.
10. Grijalva- Eternod CS, Kerac M, McGrath M, et al. Admission profile and discharge outcomes for infants aged less than 6 months admitted to inpatient therapeutic care in 10 countries. A secondary data analysis. *Maternal & child nutrition* 2017; **13**(3): e12345.
11. Campion-Smith TJ, Kerac M, McGrath M, Berkley JA. Antimicrobial and micronutrient interventions for the management of infants under 6 months of age identified with severe malnutrition: a literature review. *PeerJ* 2020; **8**: e9175.
12. Black RE, Victora CG, Walker SP, et al. Maternal and child undernutrition and overweight in low-income and middle-income countries. *Lancet* 2013; **382**(9890): 427-51.
13. Han Z, Lutsiv O, Mulla S, McDonald SD. Maternal height and the risk of preterm birth and low birth weight: a systematic review and meta-analyses. *J Obstet Gynaecol Can* 2012; **34**(8): 721-46.
14. Khatun W, Rasheed S, Alam A, Huda TM, Dibley MJ. Assessing the intergenerational linkage between short maternal stature and under-five stunting and wasting in Bangladesh. *Nutrients* 2019; **11**(8): 1818.
15. Rijken MJ, De Livera AM, Lee SJ, et al. Quantifying low birth weight, preterm birth and small-for-gestational-age effects of malaria in pregnancy: a population cohort study. *PLoS One* 2014; **9**(7): e100247.

16. Saito M, Briand V, Min AM, McGready R. Deleterious effects of malaria in pregnancy on the developing fetus: a review on prevention and treatment with antimalarial drugs. *The Lancet Child & Adolescent Health* 2020; **4**(10): 761-74.
17. Abdi F, Alimoradi Z, Alidost F. Pregnancy outcomes and effects of antiretroviral drugs in HIV-positive pregnant women: a systematic review. *Future Virology* 2019; **14**(3): 197-210.
18. Ko JY, Desisto CL, Simeone RM, et al. Adverse Pregnancy Outcomes, Maternal Complications, and Severe Illness Among US Delivery Hospitalizations With and Without a Coronavirus Disease 2019 (COVID-19) Diagnosis. *Clinical Infectious Diseases* 2021; **73**(Supplement_1): S24-S31.
19. Husseini M, Darboe MK, Moore SE, Nabwera HM, Prentice AM. Thresholds of socio-economic and environmental conditions necessary to escape from childhood malnutrition: a natural experiment in rural Gambia. *BMC Medicine* 2018; **16**(1).
20. Brander RL, Pavlinac PB, Walson JL, et al. Determinants of linear growth faltering among children with moderate-to-severe diarrhea in the Global Enteric Multicenter Study. *BMC medicine* 2019; **17**: 1-16.
21. Ross KM, Guardino C, Dunkel Schetter C, Hobel CJ. Interactions between race/ethnicity, poverty status, and pregnancy cardio-metabolic diseases in prediction of postpartum cardio-metabolic health. *Ethnicity & health* 2020; **25**(8): 1145-60.
22. Li F, Wang T, Chen L, Zhang S, Chen L, Qin J. Adverse pregnancy outcomes among mothers with hypertensive disorders in pregnancy: a meta-analysis of cohort studies. *Pregnancy Hypertension* 2021.
23. Relph S, Patel T, Delaney L, Sobhy S, Thangaratinam S. Adverse pregnancy outcomes in women with diabetes-related microvascular disease and risks of disease progression in pregnancy: A systematic review and meta-analysis. *PLOS Medicine* 2021; **18**(11): e1003856.
24. Kaldenbach S, Engebretsen IM, Haskins L, Conolly C, Horwood C. Infant feeding, growth monitoring and the double burden of malnutrition among children aged 6 months and their mothers in KwaZulu- Natal, South Africa. *Maternal & Child Nutrition* 2022; **18**(1): e13288.
25. Pérez-Escamilla R. Food security and the 2015–2030 sustainable development goals: From human to planetary health: Perspectives and opinions. *Current developments in nutrition* 2017; **1**(7): e000513.
26. Fleischer NL, Merialdi M, van Donkelaar A, et al. Outdoor air pollution, preterm birth, and low birth weight: analysis of the world health organization global survey on maternal and perinatal health. *Environmental health perspectives* 2014; **122**(4): 425-30.
27. Yuan L, Zhang Y, Gao Y, Tian Y. Maternal fine particulate matter (PM2.5) exposure and adverse birth outcomes: an updated systematic review based on cohort studies. *Environmental Science and Pollution Research* 2019; **26**(14): 13963-83.
28. Tusting LS, Bradley J, Bhatt S, et al. Environmental temperature and growth faltering in African children: a cross-sectional study. *The Lancet Planetary Health* 2020; **4**(3): e116-e23.
29. Dadi AF, Miller ER, Mwanri L. Postnatal depression and its association with adverse infant health outcomes in low- and middle-income countries: a systematic review and meta-analysis. *BMC Pregnancy Childbirth* 2020; **20**(1): 416.
30. Surkan PJ, Patel SA, Rahman A. Preventing infant and child morbidity and mortality due to maternal depression. *Best Pract Res Clin Obstet Gynaecol* 2016; **36**: 156-68.

31. Surkan PJ, Kennedy CE, Hurley KM, Black MM. Maternal depression and early childhood growth in developing countries: systematic review and meta-analysis. *Bull World Health Organ* 2011; **89**(8): 608-15.
32. Lawrence RM, Lawrence RA. Normal Growth, Growth Faltering, and Obesity in Breastfed Infants. Breastfeeding: Elsevier; 2022: 298-325.
33. Ghanchi A, Derridj N, Bonnet D, Bertille N, Salomon LJ, Khoshnood B. Children Born with Congenital Heart Defects and Growth Restriction at Birth: A Systematic Review and Meta-Analysis. *Int J Environ Res Public Health* 2020; **17**(9).
34. Mekonnen AG, Odo DB, Nigatu D, Sav A, Abagero KK. Women's empowerment and child growth faltering in Ethiopia: evidence from the Demographic and Health Survey. *BMC Womens Health* 2021; **21**(1).
35. Na M, Jennings L, Talegawkar SA, Ahmed S. Association between women's empowerment and infant and child feeding practices in sub-Saharan Africa: an analysis of Demographic and Health Surveys. *Public health nutrition* 2015; **18**(17): 3155-65.
36. von Salmuth V, Brennan E, Kerac M, McGrath M, Frison S, Lelijveld N. Maternal-focused interventions to improve infant growth and nutritional status in low-middle income countries: A systematic review of reviews. *PLoS One* 2021; **16**(8): e0256188.
37. Pérez-Escamilla R, Hromi-Fiedler A, Rhodes E, et al. Impact of prelacteal feeds and neonatal introduction of breastmilk substitutes on breastfeeding outcomes: A systematic review and meta-analysis. *Matern Child Nutr* 2022; **18**: e13368.
38. Vilar- Compte M, Pérez- Escamilla R, Orta- Aleman D, et al. Impact of baby behaviour on caregiver's infant feeding decisions during the first 6 months of life: A systematic review. *Maternal & Child Nutrition* 2022; **18**(S3): e13345.
39. Martin A, Connelly A, Bland RM, Reilly JJ. Health impact of catch-up growth in low-birth weight infants: systematic review, evidence appraisal, and meta-analysis. *Matern Child Nutr* 2017; **13**(1).
40. Amugsi DA, Dimbuene ZT. Basic determinants of child linear growth outcomes in sub-Saharan Africa: a cross-sectional survey analysis of positive deviants in poor households. *Scientific Reports* 2022; **12**(1): 14218.
41. Organization. WH. WHO recommendations for care of the preterm or low-birth-weight infant. Geneva, Switzerland: World Health Organization, 2022.
42. DiLauro S, Unger S, Stone D, O'Connor DL. Human milk for ill and medically compromised infants: strategies and ongoing innovation. *Journal of Parenteral and Enteral Nutrition* 2016; **40**(6): 768-82.
43. Aneja S, Kumar P, Choudhary TS, et al. Growth faltering in early infancy: highlights from a two-day scientific consultation. BMC proceedings; 2020: Springer; 2020. p. 1-7.
44. Sarkis-Onofre R, Catalá-López F, Aromataris E, Lockwood C. How to properly use the PRISMA Statement. *Systematic Reviews* 2021; **10**(1): 1-3.
45. Sterne J, Savović J, Page M, et al. RoB 2: a revised tool for assessing risk of bias in randomised trials. . *BMJ* 2019; **366**: l4898.
46. Alonso-Coello P, Schünemann HJ, Moberg J, et al. GRADE Evidence to Decision (EtD) frameworks: a systematic and transparent approach to making well informed healthcare choices. 1: Introduction. *bmj* 2016; **353**.
47. Clarke SE, Evans S, Macdonald A, Davies P, Booth IW. Randomized comparison of a nutrient-dense formula with an energy-supplemented formula for infants with faltering growth. *J Hum Nutr Diet* 2007; **20**(4): 329-39.

48. Evans S, Twaissi H, Daly A, Davies P, Macdonald A. Should high-energy infant formula be given at full strength from its first day of usage? *J Hum Nutr Diet* 2006; **19**(3): 191-7; quiz 9.

49. Islam MM, Huq S, Hossain MI, et al. Efficacy of F-100, diluted F-100, and infant formula as rehabilitation diet for infants aged < 6 months with severe acute malnutrition: a randomized clinical trial. *Eur J Nutr* 2020; **59**(5): 2183-93.

50. Wilkinson C. Comparative study of the effectiness of infant formula and diluted F-100 therapeutic milk products in the treatment of severe acute malnutrition in infants under six months of age: Technical Department Research Service ACF-France, 2008.

51. Vesel L, Bellad RM, Manji K, et al. Feeding practices and growth patterns of moderately low birthweight infants in resource-limited settings: results from a multisite, longitudinal observational study. *BMJ open* 2023; **13**(2): e067316.

52. Mohandas S, Rana R, Sirwani B, Kirubakaran R, Puthussery S. Effectiveness of Interventions to Manage Difficulties with Breastfeeding for Mothers of Infants under Six Months with Growth Faltering: A Systematic Review Update. *Nutrients* 2023; **15**(4): 988.

53. Clinical Trials Registry - India CTRI/2022/07/043716. Nutritional management of growth faltering in infants aged under six months in Asia and Africa. 2022. http://www.ctri.nic.in/Clinicaltrials/pmaindet2.php?trialid=70345 (accessed November 19 2022).

54. Gonzalez Garay AG, Medina Vera I, Serralde-Zúñiga AE, Velasco Hidalgo L, Alonso Ocaña MV. Higher versus lower protein intake in formula- fed term infants. *Cochrane Database of Systematic Reviews* 2020; (10).

55. Pereira-Da-Silva L, Virella D, Fusch C. Nutritional Assessment in Preterm Infants: A Practical Approach in the NICU. *Nutrients* 2019; **11**(9): 1999.

56. Grey K, Brennan E, Kerac M, McGrath M. The MAMI Care Pathway Package: A resource to support the management of small and nutritionally at-risk infants under six months of age and their mothers (MAMI). *South Sudan Medical Journal* 2021; **14**(3): 94-7.

57. World Health Organization. WHO guideline on the prevention and management of wasting and nutritional oedema (acute malnutrition) in infants and children under 5 years Geneva, Switzerland: World Health Organization, 2023.

58. Pérez-Escamilla R, Bermudez O, Buccini GS, et al. Nutrition disparities and the global burden of malnutrition. *BMJ* 2018; **361**: k2252.

Figure

Click here to access/download;Figure;Figure 1 PRISMA flow chart 8-10-23.tiff

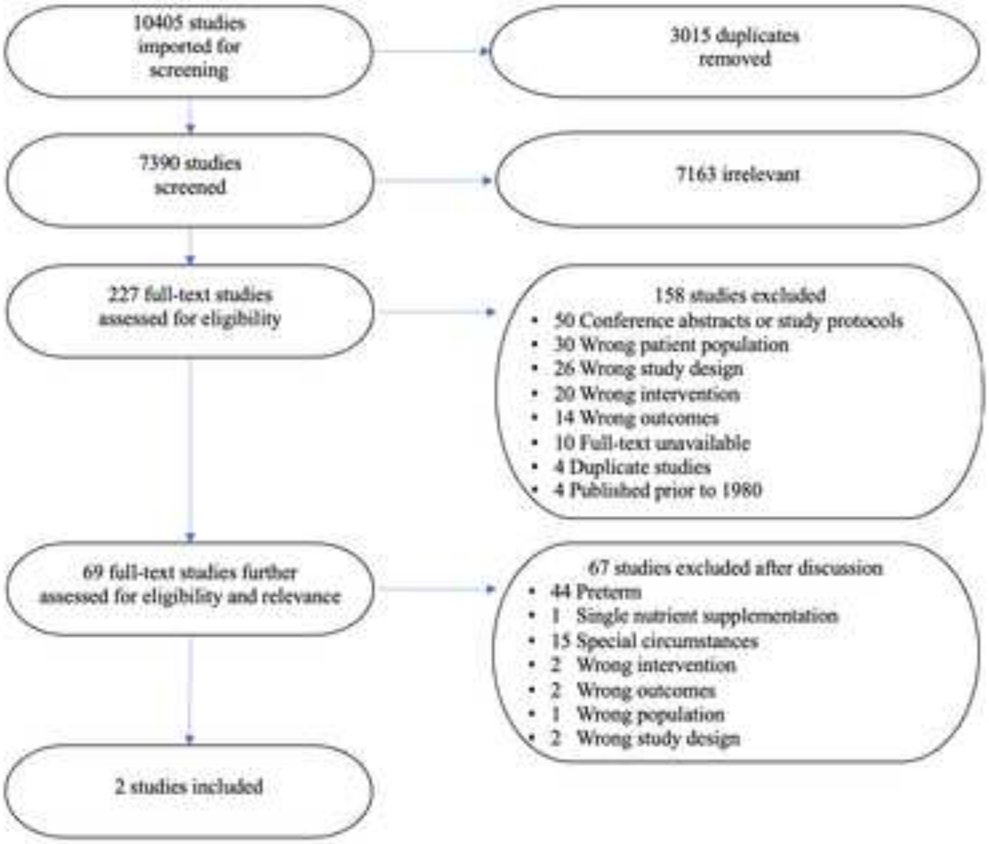

Figure

Click here to access/download;Figure;Figure 2. Metaanalysis of weight gain 8-10-23.tiff

Study name	Statistics for each study				
	Difference in means	Lower limit	Upper limit	Z-Value	p-Value
Islam et al (2020)	3.10	0.68	5.52	2.51	0.012
Wilkinson (2008)	-0.10	-3.46	3.26	-0.06	0.953
	1.72	-1.39	4.83	1.09	0.278

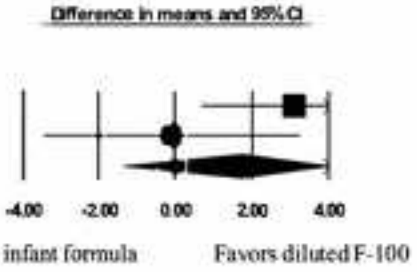

Figure

Click here to access/download;Figure;Figure 3. Research framework.tiff

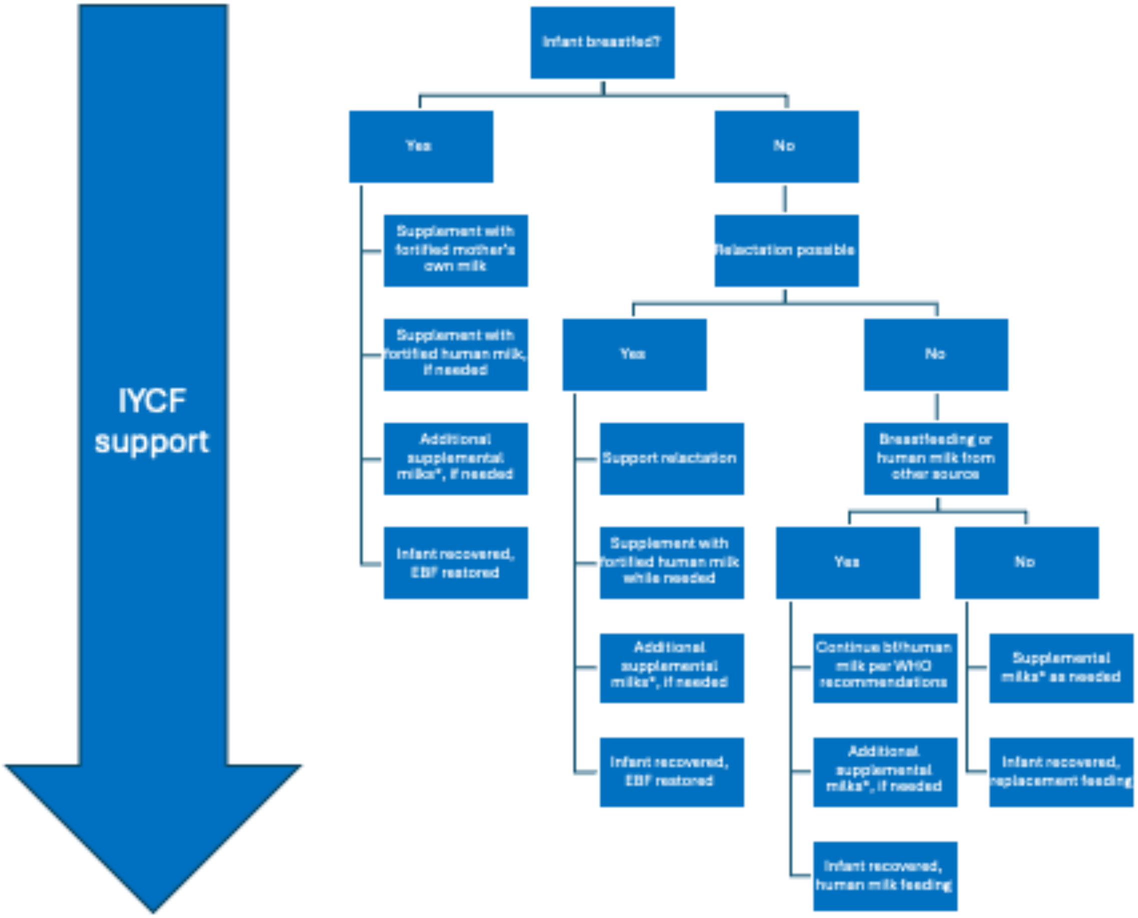

Figure

Click here to access/download;Figure;Table 1 Summary of included studies 8-10-23.tiff

First author, year. Location/ setting	Design	Sample description	Interventions	Outcomes	Risk of bias
**Islam, 2019 Dhaka, Bd Nutrition rehabilitation unit** <br>**2012-2015**	RCT, double blinded	n=153 infants with diarrheal illness. <6mos, weight-for-length Z-score<-3 and/or bipedal edema. After stabilization: free of edema with no clinical signs of infection.	After stabilization phase with F-75, randomized to 130 ml/kg/day in 12 (2-hourly) feeds, and then 10 ml more at each feed for the first 2 days and then fed at 3-hourly intervals: **Intx. 1**: F-100 (n=50) **Intx. 2** Diluted F-100 (n=52) **Intx. 3** Infant formula. (n=51) Mothers who breastfed their hospitalized infants were encouraged to continue. If infant became ill→transferred to specialty care ward and fed F-75 instead of assigned diet. After recovery, resumed allocated diet.	Main: Rate of weight gain (g/kg/day) in rehabilitation phase. Secondary: potential renal solute load (PRSL (mOmsmol/l), renal solute load (RSL) (mOsmol/l), serum sodium (mmol/l), urinary specific gravity; study diet intake (g/day) and (kcal/day); breast milk intake (if the child is breastfed) (g/d) and (kcal/d), duration of stay at the NRU (in days)	High
**Wilkinson, 2008 South Kivu, DRC 2007-2008**	RCT	n= 146 infants. <6mos at admission, currently breastfed, with failure of effective breastfeeding, or infant too weak to suckle effectively, or infant is not gaining weight at home. No oedema, or other conditions that interfered with ability to breastfeeding.	Supplementary quantities of artificial milk were given every three hours: Control: Diluted F100 Intervention: Generic infant formula for children 0-12 months. Frequent breastfeeding and/or expressed breastmilk was encouraged for all infants	Primary: rate of weight gain (g/kg/day) and mortality. Secondary: weight gain (g), duration of treatment (days), recovery, and defaulting.	High

Click here to access/download;Figure;Table 2. DF100-vs-IF 3-21-23 8-10-23_Page_1.tiff

Certainty assessment							№ of patients		Effect		Certainty	Importance
№ of studies	Study design	Risk of bias	Inconsistency	Indirectness	Imprecision	Other considerations	diluted F-100	infant formula	Relative (95% CI)	Absolute (95% CI)		
**Rate of weight gain (g/kg/day), during the rehabilitation phase (Islam 2013) or from admission until discharge in infants who recovered (Wilkinson 2008)**												
2	randomised trials	serious	not serious	serious	serious	none	100	101	-	MD **1.72 higher** (1.19 lower to 4.63 higher)	⨁◯◯◯ Very low	CRITICAL
**Body weight (kg), at the end of the rehabilitation phase, total duration of stay unclear (Islam 2013)**												
1	randomised trials	serious	not serious	serious	serious	none	52	51	-	MD **0.08 higher** (0.29 lower to 0.45 higher)	⨁◯◯◯ Very low	CRITICAL
**Length (cm), at the end of the rehabilitation phase, total duration of stay unclear (Islam 2013)**												
1	randomised trials	serious	not serious	serious	not serious	none	52	51	-	MD **0.3 lower** (2.03 lower to 1.43 higher)	⨁⨁◯◯ Low	CRITICAL
**WLZ, at the end of the rehabilitation phase, total duration of stay unclear (Islam 2013)**												
1	randomised trials	serious	not serious	serious	serious	none	48	49	-	MD **0.3 higher** (0.06 lower to 0.69 higher)	⨁◯◯◯ Very low	CRITICAL
**WAZ, at the end of the rehabilitation phase, total duration of stay unclear (Islam 2013)**												
1	randomised trials	serious	not serious	serious	serious	none	50	49	-	MD **0.1 lower** (0.4 lower to 0.6 higher)	⨁◯◯◯ Very low	CRITICAL
**LAZ, at the end of the rehabilitation phase, total duration of stay unclear (Islam 2013)**												
1	randomised trials	serious	not serious	serious	serious	none	52	51	-	MD **0** (0.05 lower to 0.05 higher)	⨁◯◯◯ Very low	CRITICAL
**Mortality (Islam 2013, Wilkinson 2008)**												
2	randomised trials	serious	not serious	serious	serious	none	12/126 (9.5%)	11/123 (8.9%)	**RR 1.06** (0.49 to 2.32)	**5 more per 1,000** (from 46 fewer to 116 more)	⨁◯◯◯ Very low	IMPORTANT

Clinical deterioration defined by development of any danger signs - not measured

Click here to access/download;Figure;Table 2. DF100-vs-IF 3-21-23 8-10-23_Page_2.tiff

Certainty assessment							№ of patients		Effect		Certainty	Importance
№ of studies	Study design	Risk of bias	Inconsistency	Indirectness	[illegible]precision	Other considerations	diluted F-100	infant formula	Relative (95% CI)	Absolute (95% CI)		
-	-	-	-	-	-	-	-	-	-	-	-	IMPORTANT
**Morbidity or recovery from co-morbidity: "hospital-acquired infection" (Islam 2019)**												
[illegible]	randomised trials	serious[illegible]	not serious	serious[illegible]	serious[illegible]	none	20/52 (38.5%)	13/51 (25.5%)	**RR 1.51** (0.84 to 2.70)	**130 more per 1,000** (from 41 fewer to 433 more)	⊕◯◯◯ Very low	IMPORTANT
**Relapse - not measured**												
-	-	-	-	-	-	-	-	-	-	-	-	IMPORTANT
**Readmission - not measured**												
-	-	-	-	-	-	-	-	-	-	-	-	IMPORTANT
**Non-response - not measured**												
-	-	-	-	-	-	-	-	-	-	-	-	IMPORTANT
**Potential renal solute load (mOsm/day), at discharge (Islam 2019)**												
1	randomised trials	serious[illegible]	not serious	serious[illegible]	serious[illegible]	none	52	51	-	MD **21.5 higher** (6.4 higher to 36.5 higher)	⊕◯◯◯ Very low	
**Renal solute load (mOsm/day), at discharge (Islam 2019)**												
1	randomised trials	serious[illegible]	not serious	serious[illegible]	serious[illegible]	none	52	51	-	MD **16.2 higher** (1.5 higher to 30.8 higher)	⊕◯◯◯ Very low	
**Serum sodium (mmol/L), day 7 (Islam 2019)**												
1	randomised trials	serious[illegible]	not serious	serious[illegible]	not serious	none	44	41	-	MD **0.3 lower** (1.3 lower to 0.7 higher)	⊕⊕◯◯ Low	
**Serum chloride (mmol/L), day 7 (Islam 2019)**												
1	randomised trials	serious[illegible]	not serious	serious[illegible]	not serious	none	44	41	-	MD **0.2 higher** (1.21 lower to 1.61 higher)	⊕⊕◯◯ Low	

CI: confidence interval; **MD:** mean difference; **RR:** risk ratio

Explanations

Click here to access/download;Figure;Table 2. DF100-vs-IF 3-21-23 8-10-23_Page_3.tiff

a. One study (Islam 2019) included infants under six months admitted for diarrheal illness with weight-for-length z score of <-3 and/or bilateral oedema that were stabilized in less than 9 days (free of edema, with cessation of vomiting, fewer than 3 watery stools /day and no clinical signs of infection). However, if any of the infants became ill, they were transferred back to a Longer Stay or Special Care Ward and fed F-75, after recovery they were returned to the study diet. The other study (Wilkinson 2008) included infants under six months who were too weak or feeble to suckle effectively, with breastfeeding failure, not gaining weight at home, or with weight-for-length below 70% of the NCHS median. Infants with oedema were excluded.

b. One study (Islam 2019) fed infants 2 hourly F75 during stabilization phase. For the first 2 days of rehabilitation phase they received 130 ml/kg/day of F100 or infant formula in 12 (2 hr) feeds and then 10 ml more at each feed until some was left (ad libitum). Water was freely available. The other study (Wilkinson 2008) gave supplementary quantities of artificial milk every 3 hours, using the SST technique, frequent breastfeeding was encouraged. When infants gained >=20g/day, for 3 consecutive days, the milk supplement was reduced to 1/2, when infants maintained weight gain >=10g/day on reduced quantities for 3 consecutive days, milk supplement was stopped altogether and infant received only breast milk, when infants maintained weight gain of 10g/day for three to three to five consecutive days, the infant considered recovered and discharged.

c. Serious risk of bias. One of the two studies (Wilkinson 2008) had a high risk of bias (due to unblinding, loss to follow-up) while the other study (Islam 2019) had a high risk of bias (due to non-adherence to the assigned intervention, loss to follow-up).

d. Serious indirectness. The supplemental [illegible] the Islam 2019 trial were given only to infants with severe wasting and/or oedema who had already been stabilized and given F-75 prior to the rehabilitation phase, and if they became ill they were given F-75 again before resuming one of the three study milks. See footnotes [illegible] for additional details of the study populations and intervention approaches.

e. Serious imprecision. 95% CI around the absolute effect crosses the null, and the effect ranges from trivial harm to moderate benefit.

f. Serious risk of bias. The only study (Islam 2019) had had a high risk of bias (due to non-adherence to the assigned intervention, loss to follow-up).

g. Serious imprecision. 95% CI around the absolute effect crosses the null, and the effect ranges from small harm to small benefit.

h. Serious imprecision. 95% CI around the absolute effect crosses the null, and the effect ranges from trivial harm to small benefit.

i. Serious imprecision. 95% CI around the absolute effect crosses the null, and the effect ranges from small benefit to moderate harm.

j. "Fifty-two infants temporarily went to the Longer Stay Ward or Special Care Ward for management of hospital-acquired infection during the intervention period. The numbers were 19, 20 and 13, respectively, in the F-100, diluted F-100, and infant formula groups (P= 0.29)."

k. Serious imprecision. 95% CI around the absolute effect crosses the null, and the effect ranges from small benefit to serious harm.

l. Serious imprecision. 95% CI around the absolute effect does not cross the null threshold, however the 95% CI includes both small to moderate harm.

Click here to access/download;Figure;Table 3. F100-vs-IF 8-10-23_Page_1.tiff

Certainty assessment							№ of patients		Effect		Certainty	Importance
№ of studies	Study design	Risk of bias	Inconsistency	Indirectness	Imprecision	Other considerations	F-100	infant formula	Relative (95% CI)	Absolute (95% CI)		
**Rate of weight gain (g/kg/day), during the rehabilitation phase (Islam 2013)**												
1	randomised trials	serious[a]	not serious	serious[b]	serious[c]	none	50	51	-	MD **4.6 higher** (1.5 higher to 7.6 higher)	⊕◯◯◯ Very low	CRITICAL
**Body weight (kg), at the end of the rehabilitation phase, total duration of stay unclear (Islam 2013)**												
1	randomised trials	serious[a]	not serious	serious[b]	serious[c]	none	50	51	-	MD **0.08 lower** (0.43 lower to 0.27 higher)	⊕◯◯◯ Very low	CRITICAL
**Length (cm), at the end of the rehabilitation phase, total duration of stay unclear (Islam 2013)**												
1	randomised trials	serious[a]	not serious	serious[b]	not serious	none	50	51	-	MD **0.4 lower** (1.95 lower to 1.15 higher)	⊕⊕◯◯ Low	CRITICAL
**WLZ, at the end of the rehabilitation phase, total duration of stay unclear (Islam 2013)**												
1	randomised trials	serious[a]	not serious	serious[b]	serious[c]	none	49	49	-	MD **0.1 lower** (0.58 lower to 0.38 higher)	⊕◯◯◯ Very low	CRITICAL
**WAZ, at the end of the rehabilitation phase, total duration of stay unclear (Islam 2013)**												
1	randomised trials	serious[a]	not serious	serious[b]	serious[c]	none	49	49	-	MD **0.1 lower** (0.6 lower to 0.4 higher)	⊕◯◯◯ Very low	CRITICAL
**LAZ, at the end of the rehabilitation phase, total duration of stay unclear (Islam 2013)**												
1	randomised trials	serious[a]	not serious	serious[b]	serious[c]	none	52	51	-	MD **0** (0.61 lower to 0.61 higher)	⊕◯◯◯ Very low	CRITICAL
**Mortality (Islam 2013)**												
1	randomised trials	serious[a]	not serious	serious[b]	not serious	none	1/50 (2.0%)	0/51 (0.0%)	**RR 3.06** (0.13 to 73.28)	**4 more per 1,000** (from 2 lower to 142 more)	⊕◯◯◯ Very low	IMPORTANT

Clinical deterioration defined by development of any danger signs - not measured

Click here to access/download;Figure;Table 3. F100-vs-IF 8-10-23_Page_2.tiff

Certainty assessment							№ of patients		Effect		Certainty	Importance
№ of studies	Study design	Risk of bias	Inconsistency	Indirectness	Imprecision	Other considerations	F-100	infant formula	Relative (95% CI)	Absolute (95% CI)		
-	-	-	-	-	-	-	-	-	-	-	-	IMPORTANT
**Morbidity or recovery from co-morbidity: "hospital-acquired infection" (Islam 2019)**												
1	randomised trials	serious[a]	not serious	serious[a]	serious[b]	none	10/50 (20.0%)	13/51 (25.5%)	**RR 1.49** (0.83 to 2.68)	**125 more per 1,000** (from 43 fewer to 420 more)	⊕◯◯◯ Very low	IMPORTANT
**Relapse - not measured**												
-	-	-	-	-	-	-	-	-	-	-	-	IMPORTANT
**Readmission - not measured**												
-	-	-	-	-	-	-	-	-	-	-	-	IMPORTANT
**Non-response - not measured**												
-	-	-	-	-	-	-	-	-	-	-	-	IMPORTANT
**Potential renal solute load (mOsm/day), at discharge (Islam 2019)**												
1	randomised trials	serious[a]	not serious	serious[a]	serious[b]	none	50	51	-	MD **44.6 higher** (27.2 higher to 62.1 higher)	⊕◯◯◯ Very low	
**Renal solute load (mOsm/day), at discharge (Islam 2019)**												
1	randomised trials	serious[a]	not serious	serious[a]	serious[b]	none	50	51	-	MD **40.2 higher** (23.5 higher to 57 higher)	⊕◯◯◯ Very low	
**Serum sodium (mmol/L), day 7 (Islam 2019)**												
1	randomised trials	serious[a]	not serious	serious[a]	not serious	none	36	41	-	MD **0.6 lower** (1.7 lower to 0.5 lower)	⊕⊕◯◯ Low	
**Serum chloride (mmol/L), day 7 (Islam 2019)**												
1	randomised trials	serious[a]	not serious	serious[a]	not serious	none	36	41	-	MD **1.4 higher** (0.26 lower to 3.06 higher)	⊕⊕◯◯ Low	

CI: confidence interval; **MD:** mean difference; **RR:** risk ratio

Explanations

Click here to access/download;Figure;Table 3. F100-vs-IF 8-10-23_Page_3.tiff

a. Serious risk of bias: The only study (Islam 2019) had had a high risk of bias (due to non adherence to the assigned intervention, loss to follow up)

b. Serious indirectness: The supplemental milks in the Islam 2019 trial were given only to infants with severe wasting and/or oedema who had already been stabilized and given F-75 prior to the rehabilitation phase, and if they became ill they were given F-75 again before resuming one of the three study milks. See footnotes a and b for additional details of the study populations and intervention approaches

c. Serious imprecision: 95% CI around the absolute effect does not cross the null threshold, however the 95% CI includes both small to moderate benefit

d. Serious imprecision: 95% CI around the absolute effect crosses the null, and the effect ranges from trivial harm to trivial benefit

e. Serious imprecision: 95% CI around the absolute effect crosses the null, and the effect ranges from small harm to trivial benefit

f. Serious imprecision: 95% CI around the absolute effect crosses the null, and the effect ranges from small benefit to serious harm.

g. "Fifty-two infants temporarily went to the Longer Stay Ward or Special Care Ward for management of hospital-acquired infection during the intervention period. The numbers were 19, 20 and 13, respectively, in the F-100, diluted F-100, and infant formula groups (P = 0.29)."

h. Serious imprecision: 95% CI around the absolute effect crosses the null, and the effect ranges from small benefit to serious harm

i. Serious imprecision: 95% CI around the absolute effect does not cross the null threshold, however the 95% CI includes both small to moderate harm.

Click here to access/download;Figure;Table 4. F100-vs-DF100 8-10-23_Page_1.tiff

Certainty assessment							№ of patients		Effect		Certainty	Importance
№ of studies	Study design	Risk of bias	Inconsistency	Indirectness	Imprecision	Other considerations	F-100	diluted F-100	Relative (95% CI)	Absolute (95% CI)		
**Rate of weight gain (g/kg/day), during the rehabilitation phase (Islam 2013)**												
1	randomised trials	serious [a]	not serious	serious [b]	serious [c]	none	50	52	-	MD **1.5 higher** (1.7 lower to 4.8 higher)	⊕◯◯◯ Very low	CRITICAL
**Body weight (kg), at the end of the rehabilitation phase, total duration of stay unclear (Islam 2013)**												
1	randomised trials	serious [a]	not serious	serious [b]	serious [c]	none	50	52	-	MD **0.16 lower** (0.57 lower to 0.25 higher)	⊕◯◯◯ Very low	CRITICAL
**Length (cm), at the end of the rehabilitation phase, total duration of stay unclear (Islam 2013)**												
1	randomised trials	serious [a]	not serious	serious [b]	not serious	none	50	52	-	MD **0.1 lower** (1.36 lower to 1.76 higher)	⊕⊕◯◯ Low	CRITICAL
**WLZ, at the end of the rehabilitation phase, total duration of stay unclear (Islam 2013)**												
1	randomised trials	serious [a]	not serious	serious [b]	serious [c]	none	49	48	-	MD **0.4 lower** (0.83 lower to 0.03 higher)	⊕◯◯◯ Very low	CRITICAL
**WAZ, at the end of the rehabilitation phase, total duration of stay unclear (Islam 2013)**												
1	randomised trials	serious [a]	not serious	serious [b]	serious [c]	none	49	50	-	MD **0.2 lower** (0.75 lower to 0.35 higher)	⊕◯◯◯ Very low	CRITICAL
**LAZ, at the end of the rehabilitation phase, total duration of stay unclear (Islam 2013)**												
1	randomised trials	serious [a]	not serious	serious [b]	serious [c]	none	50	52	-	MD **0** (0.74 lower to 0.74 higher)	⊕◯◯◯ Very low	CRITICAL
**Mortality (Islam 2013)**												
1	randomised trials	serious [a]	not serious	serious [b]	not serious	none	1/50 (2.0%)	0/52 (0.0%)	**RR 3.12** (0.13 to 74.75)	**4 more per 1,000** (from 2 lower to 142 more)	⊕◯◯◯ Very low	IMPORTANT

Clinical deterioration defined by development of any danger signs - not measured

Click here to access/download;Figure;Table 4. F100-vs-DF100 8-10-23_Page_2.tiff

Certainty assessment							№ of patients		Effect		Certainty	Importance
№ of studies	Study design	Risk of bias	Inconsistency	Indirectness	Imprecision	Other considerations	F-100	diluted F-100	Relative (95% CI)	Absolute (95% CI)		
-	-	-	-	-	-	-	-	-	-	-	-	IMPORTANT
**Morbidity or recovery from co-morbidity: "hospital-acquired infection" (Islam 2019)**												
1	randomised trials	serious[illegible]	not serious	serious[illegible]	serious[illegible]	none	19/50 (38.0%)	20/52 (38.5%)	**RR 0.99** (0.60 to 1.62)	**4 fewer per 1,000** (from 354 fewer to 220 more)	⨁◯◯◯ Very low	IMPORTANT
**Relapse - not measured**												
-	-	-	-	-	-	-	-	-	-	-	-	IMPORTANT
**Readmission - not measured**												
-	-	-	-	-	-	-	-	-	-	-	-	IMPORTANT
**Non-response - not measured**												
-	-	-	-	-	-	-	-	-	-	-	-	IMPORTANT
**Potential renal solute lo[illegible]mOsm/day), at discharge (Islam 2019)**												
1	randomised trials	serious[illegible]	not serious	serious[illegible]	serious[illegible]	none	50	52	-	MD **23.1 higher** (4.1 higher to 42.2 higher)	⨁◯◯◯ Very low	
**Renal solute load (mOsm/day), at discharge (Islam 2019)**												
1	randomised trials	serious[illegible]	not serious	serious[illegible]	serious[illegible]	none	50	52	-	MD **24 higher** (5.2 higher to 42.9 higher)	⨁◯◯◯ Very low	
**Serum sodium (mmol/L), day 7 (Islam 2019)**												
1	randomised trials	serious[illegible]	not serious	serious[illegible]	not serious	none	36	44	-	MD **0.2 lower** (1.07 lower to 0.67 higher)	⨁⨁◯◯ Low	
**Serum chloride (mmol/L), day 7 (Islam 2019)**												
1	randomised trials	serious[illegible]	not serious	serious[illegible]	not serious	none	36	44	-	MD **1.2 higher** (0.27 lower to 2.67 higher)	⨁⨁◯◯ Low	

CI: confidence interval; **MD:** mean difference; **RR:** risk ratio

Explanations

Click here to access/download;Figure;Table 4. F100-vs-DF100 8-10-23_Page_3.tiff

a. Serious risk of bias: The only study (Islam 2019) had had a high risk of bias (due to non-adherence to the assigned intervention, loss to follow up).

b. Serious indirectness: The supplemental milks in the Islam 2019 trial were given only to infants with severe wasting and/or oedema who had already been stabilized and given F-75 prior to the rehabilitation phase, and if they became ill they were given F-75 again before resuming one of the three study milks. See footnotes a and b for additional details of the study populations and intervention approaches.

c. Serious imprecision: 95% CI around the absolute effect crosses the null, and the effect ranges from trivial harm to small benefit.

d. Serious imprecision: 95% CI around the absolute effect crosses the null, and the effect ranges from trivial harm to trivial benefit.

e. Serious imprecision: 95% CI around the absolute effect crosses the null, and the effect ranges from small harm to trivial benefit.

f. Serious imprecision: 95% CI around the absolute effect crosses the null, and the effect ranges from small benefit to serious harm.

g. "Fifty-two infants temporarily went to the Longer Stay Ward or Special Care Ward for management of hospital-acquired infection during the intervention period. The numbers were 19, 20 and 13, respectively, in the F-100, diluted F-100, and infant formula groups (P=0.29)."

h. Serious imprecision: 95% CI around the absolute effect crosses the null, and the effect ranges from moderate harm to moderate benefit.

i. Serious imprecision: 95% CI around the absolute effect does not cross the null threshold, however the 95% CI includes both small to moderate harm.

j. Serious imprecision: 95% CI around the absolute effect does not cross the null threshold, however the 95% CI includes both small to moderate harm.

Click here to access/download
Supporting Information
Appendix 1 Search strategy.pdf

Click here to access/download
Supporting Information
Appendix 2 Excluded studies 11-21-22.pdf

Click here to access/download
Supporting Information
PRISMA_2020_checklist 8-5-23.docx

Formatted: Numbering: Continuous

Critical Research Gaps in Treating Growth Faltering in Infants under 6 Months: A Systematic Review and Meta-Analysis

Cecília Tomori, PhD[1, 2]; Deborah L O'Connor, PhD, RD[3]; Mija Ververs, MMed, MPH, RD[2]; Dania Orta-Aleman, MPH[2]; Katerina Paone[4]; Chakra Budhathoki[1], PhD; Rafael Pérez-Escamilla, PhD[4]

[1] Johns Hopkins University School of Nursing, Baltimore, MD, USA
[2] Johns Hopkins University Bloomberg School of Public Health, Baltimore, MD, USA
[3] University of Toronto, Department of Nutritional Sciences, Temerty Faculty of Medicine, Toronto, Ontario, Canada
[4] Yale University School of Public Health, New Haven, CT, USA

Abstract

Introduction
In 2020, 149.2 million children worldwide under 5 years suffered from stunting, and 45.4 million experienced wasting. Many infants are born already stunted, while others are at high risk for growth faltering early after birth. Growth faltering is linked to transgenerational impacts of poverty and marginalization. Few interventions address growth faltering in infants under 6 months, despite a likely increasing prevalence due to the negative global economic impacts of the COVID-19 pandemic. Breastfeeding is a critical intervention to alleviate malnutrition and improve child health outcomes, but rarely receives adequate attention in growth faltering interventions.

Methods
A systematic review and meta-analysis were undertaken to identify and evaluate interventions addressing growth faltering among infants under 6 months that employed supplemental milks. The review was carried out following guidelines from the USA National Academy of Medicine.

Results
A total of 10,405 references were identified, and after deduplication 7390 studies were screened for eligibility. Of these, 227 were assessed for full text eligibility and relevance. Two randomized controlled trials were ultimately included, which differed in inclusion criteria and methodology and had few shared outcomes. Both studies had small sample sizes, high attrition and high risk of bias. A Bangladeshi study (n=153) found significantly higher rates of weight gain for F-100 and diluted F-100 (DF-100) compared with infant formula (IF), while a DRC trial (n=146) did not find statistically significant differences in rate of weight gain for DF-100 compared with IF offered in the context of broader lactation and relactation support. The meta-analysis of rate of weight gain showed no statistical difference and some evidence of moderate heterogeneity.

Conclusion
Few interventions address growth faltering among infants under 6 months. These studies have limited generalizability and have not comprehensively supported lactation. Greater investment is

necessary to accelerate research that addresses growth faltering following a new research framework that calls for comprehensive lactation support.

Introduction

A substantial portion of infants face growth faltering each year across the globe[1-4]. While definitions vary, for the purpose of this paper the term growth faltering refers to a slower rate of weight, length or head circumference gain than expected for a children's age and sex [5]. Of the 149 million children globally under 5 years who are stunted (length-for-age z score less than 2 standard deviation scores below the World Health Organization (WHO) child growth standards median[6]), recent research indicates that 15% are born stunted, and an additional 25% become stunted during the first 6 months after birth[7]. Additionally, 45.4 million children under 5 years of age, constituting approximately 6.7% of children, experienced wasting (weight-for-length z score less than 2 standard deviation scores below the WHO child growth standards median[6])[4]. Infants who experience growth faltering and do not receive adequate nutritional interventions are at increased risk for mortality and morbidity from infectious illness and from non-communicable diseases[8-11]. The causes of growth faltering are complex and multifactorial. Preterm births (PTB), and infants born with low birth weight (LBW, <2500g), and small for gestational age (SGA, birth weight <10th percentile) constitute a substantial portion of those at risk across the globe for growth faltering[6,12]. Multiple comorbidities may increase the risk of growth faltering.

Importantly, social determinants of health (SDOH), such as poverty and marginalization, are key drivers of conditions that put infants at risk for growth faltering. For instance, mothers with short stature experience higher rates of LBW and preterm births[13]. Short stature itself is the outcome of intergenerational poverty[14]. Impoverished women are at higher risk for infectious diseases such as malaria and HIV, both causes of PTB, LBW and SGA[15-17]. COVID-19 is also associated with a higher risk of preterm birth[18] – and with inequities. Poverty also drives exposures to infectious agents in the home environment due to lack of access to proper sanitation including clean water, toilets, clean housing among other factors.[19,20] Poor and marginalized women across settings are at high risk for a number of complications during pregnancy[21] including diabetes and hypertension, which are associated with a substantial burden of adverse birth outcomes[22,23]. Importantly, as the food environment is rapidly changing in many settings, people in LMIC are often facing a double burden of malnutrition, with rising childhood obesity and increasing stunting at the same time. [24,25]Additional environmental drivers of ~~-~~PTB, LBW and SGA[26,27] include indoor and outdoor air pollution, which again disproportionately affects those in poverty. High environmental temperatures are also associated with growth faltering, which is becoming a pervasive threat with the growing impacts of the climate crisis with similarly disproportionate impacts[28]. ~~Finally, poor and marginalized women across settings are at high risk for a number of complications during pregnancy[24] including diabetes and hypertension, which are associated with a substantial burden of adverse birth outcomes[25,26].~~

Maternal mental health also plays a substantial role[29-31] in growth faltering and is itself linked to poverty and related biological and psycho-emotional life stressors including household food insecurity. Genetic anomalies and developmental conditions, such as cleft palate[32], and heart conditions[33] are also drivers of growth faltering. Some of these conditions then also lead to maternal poor mental health, so this relationship can be circular[30]. Conversely, social support is a key mitigator of adverse mental health outcomes,[30] demonstrating the importance of social dynamics in caring for faltering infants. Beyond the other social determinants of adverse birth

outcomes mentioned above, additional social drivers of growth may also entail broader gender power dynamics, with women's empowerment and agency linked to infant and child growth.[34,35]

Previous work has identified substantial gaps in the availability of interventions that effectively address growth faltering during infancy and early childhood. A key element that such interventions would need to consider is whether mothers have an enabling breastfeeding environment in which skin to skin contact, early initiation of and exclusive breastfeeding are supported[2,36]. Moreover, when mothers breastfeeding confidence is undermined and there is a lack of breastfeeding support, they are more likely to introduce supplements or end breastfeeding prematurely, increasing risk of infection[37,38], and hence growth faltering. Furthermore, even when supplementation with commercial milk formula (CMF) and/or specially formulated formulas and foods have the potential for increasing growth, there are also potential risks both from the greater velocity of catch up growth and corresponding metabolic consequences as well as from greater risk of infection [39].

Specific evidence on elements of successful growth interventions is often lacking. In large cross-sectional survey in Africa, positive maternal education (years of schooling) and prenatal visits were associated with improved linear growth even in conditions of poverty, highlighting protective factors that could be the basis of future interventions [40]. Critically, kangaroo mother care (KMC) and best breastfeeding practices are rarely fully addressed in available studies[24,36,41,42]. A recent scientific consultation addressing growth faltering in India highlighted some of these research gaps as well as the inadequate follow-up after transitioning from facility-based services to the home-setting[43]. Further strategies that are now standard of care in clinical settings in many middle- and high-income countries to preserve human milk feeding in very preterm and medically compromised infants such as increasing feeding volumes, nutrient-enriching expressed human milk with commercial fortifiers or less costing nutrient modulars (e.g. protein, calcium, phosphorus) or use of donor milk have received little consideration in the context of the late-preterm or term-born infant with growth faltering[41,42]. Additionally, feeding interventions may not be adequate on their own to address growth faltering since some of the drivers require other medical treatment and/or addressing broader environmental factors.

This systematic review was commissioned by the World Health Organization to address questions about optimal supplementation of infants under 6 months of age experiencing growth faltering to inform discussion of the guidelines on treatment of infants and young children with wasting and/or oedema. The review did not examine nutritional interventions aimed at breastfeeding mothers of growth faltering infants. The framework for the review and related recommendations are to be understood in the context of best practices for infant feeding and care, which entail appropriate protection, promotion and support for breastfeeding.

Methods

This study was a systematic review and meta-analysis following the US National Academies of Sciences, Engineering, and Medicine (NASEM) guidelines. The protocol was registered with PROSPERO (CRD42022350150). The review questions were:

1. In infants under 6 months with growth faltering, which criteria best determine if and when an infant should be given a supplemental milk (in addition to breastmilk if the infant is breastfed)?
2. In infants under 6 months with growth faltering meeting criteria in question 1, what is the most effective supplemental milk and for how long should it be given?

Supplemental milk was defined as donor human milk, fortified donor human milk, fortified expressed mothers' own milk, human milk from wet nurse, commercial infant formula, nutrient enriched commercial infant formula, F-75, F-100, or diluted F-100.

Searches were conducted to examine interventions addressing infants under 6 months with growth faltering that used supplemental milks as part of their treatment. Searches were framed as broadly as possible to capture any potentially relevant literature related to the initial questions. Primary outcomes of interest were anthropometry, mortality, clinical deterioration, morbidity, relapse, readmission, and non-response to treatment. Randomized controlled trials, cluster-randomized trials, and quasi-experimental studies/non-randomized controlled studies were included. Qualitative studies, reviews, and systematic reviews, as well as observational studies were excluded. All countries and settings were included.

Searches

The following databases was searched electronically in August 2022: MEDLINE, Embase, CINAHL, Web of Science, Cochrane Library, Global Index Medicus, State of Acute Malnutrition, Emergency Nutrition Network (Field Exchange), eLENA (WHO), UNICEF, FAO, WFP, FAQR, and ClinicalTrials.gov, ISRCTN registry, ICMJE registry, World Health Organization (WHO) International Clinical Trials Registry Platform. The initial searches were designed, tested, and run by a well-trained team that includes public health scientists with expertise in breastfeeding research and a medical librarian. The search strategy developed during preliminary searches are presented in Appendix 1. Search results were combined and de-duplicated in EndNote by the JHU Welch Library Team. Experts in the field were consulted to identify additional articles to be included.

Data Extraction

The study was carried out following the guidance of Preferred Reporting Items for Systematic Reviews and Meta-Analyses (PRISMA)[44]. Search results were exported into EndNote, de-duplicated, and uploaded into Covidence systematic review software for screening.
Two research assistants independently screened for potential inclusion of all titles and abstracts identified through the search. Any discrepancies were resolved by consensus or contacting a third author. Attempts were made to contact authors of included studies to obtain clarifications or additional data. Following title and abstract screening, full texts were independently screened for inclusion. Any disagreements were resolved through discussion or by consulting a third review author, if required. Reasons of exclusion were recorded for all the studies excluded at the stage of full text screening. The results of the search are presented using the PRISMA flow diagram.

Structured forms were used for data extraction of key variables including study characteristics and outcomes in a standardized data collection form. Two review authors independently extracted data and discrepancies were resolved through discussion until consensus had been achieved or by consulting a third reviewer, if required.

Risk of bias (quality) assessment

The updated Cochrane risks of bias tool, ROB-2, was used for randomized trials[45]. Two independent authors assessed quality of all eligible studies and disagreements were resolved by consensus or contacting a third author. For all data extraction and study quality assessments two researchers were standardized against each other with screening, data extraction and to assess the quality of each study. ~~Risk of bias was assessed for meta-analyses.~~

Data synthesis

Statistical heterogeneity was assessed using τ^2 (among study variance), I^2 (degree of inconsistency), and significance of the χ^2 (chi-square) test; and visual inspection of forest plots. Based on prior clinical knowledge, we expected clinical and methodological heterogeneity in included studies and therefore, attempted to explain any observed statistical heterogeneity. The Grading of Recommendations, Assessment, Development and Evaluations (GRADE)[46] methodology was used to assess the certainty of the evidence by outcome. Two independent authors assessed quality of all eligible studies and disagreements were resolved by consensus or contacting a third author. Evidence profiles were generated via GRADE Pro Software.

Results

A total of 10405 references were identified by the searches and imported for screening (Figure 1). Of these, 3015 duplicates were removed. 7390 studies were screened against title and abstract, 7163 studies were excluded, and 227 studies were assessed for full text eligibility. A total of 158 were excluded because the studies were not fully reported (n=50 study protocols or conference abstracts), had the wrong patient population (n=30), the wrong study design (n=26), the wrong intervention (n=20), the wrong outcomes (n=14), or were duplicates (n=4). Full texts were unavailable for 10 studies.~~, and~~ Additionally, 4 were carried out prior to 1980 after which significant changes took place in the composition of infant formulas, yielding 69 studies that were published between 1980 and August 2022, when the searches were carried out. [Figure 1 here]

Of the remaining 69 studies, 60 were excluded because they focused on: 1) preterm, predominately hospitalized infants primarily from high income countries 2) infants who received single nutrient supplementation, 3) infants with other special circumstances (e.g., recovering from cardiac surgery) (Appendix 2). Two studies within the infants with special circumstances category were examined further for relevant contextual information (Clarke et al[47] and Evans et al [48]) but were ultimately excluded because the study sample was principally comprised of infants with a significant medical condition known to elevate nutritional requirements and impact growth. Only one infant (of 30) received breastmilk in a previous feed in Evans et al, while Clarke did not report any breastfeeding or receipt of breast milk, making it likely participant infants were nearly all exclusively formula-fed in both studies during the treatment period.

Evans et al[48] enrolled 30 growth faltering infants with a median age of 14.5 weeks (range 2-43 weeks) who were receiving care at a large British specialty referral hospital, 73% (n=22) of whom had cardiac defects. They compared a ready to feed high-energy (1kcal/ml) nutrient-dense (concentrated macro- and micronutrients) infant formula at full strength (25 mOsm kg^{-1} H_2O) from day one with stepwise introduction of the same ready to feed infant formula but in a diluted form and graded to full strength over three days; infants were studied for a duration of 2 weeks. While younger infants in the high energy nutrient dense-formula group had an initial increased number of stools, no differences in feeding tolerance nor growth were observed between treatment arms.

Clarke et al[47] enrolled 60 growth faltering infants with a median age of 5 weeks (range 2-31) with cardiac lesions, cystic fibrosis, or other organic causes from Birmingham Children's Hospital in the UK. This open RCT compared two specialty infant formulas, 'high-energy nutrient-dense formula' and a 'high energy only-supplemented formula,' for a period of 6 weeks. The energy concentration of both formulas was 1 kcal/ml; energy was supplied in the "high energy only-supplemented formula" by adding glucose whereas in the "high energy nutrient-dense formula" it was supplied by protein and glucose. The protein concentration of the high energy only and high energy nutrient dense formulas was 1.4 and 2.6 grams/100 kcal, respectively. Infants in the "high energy nutrient-dense formula" group had higher intakes of protein, vitamins and minerals and blood urea nitrogen levels suggesting more favorable protein balance; further there was less fall off of length for age-z-scores compared to the "high energy only-supplemented" group.

An additional seven studies were determined to have the wrong intervention, outcomes, population, or study design (Appendix 2). While initial searches undertaken were purposefully broad, in the full text review of manuscripts it became clear that most included studies focused on preterm babies in high-income settings and/or addressed special populations with particular medical conditions. These studies did not address treatment and recovery from malnutrition, which was the purpose of this review. Therefore, after careful consideration 67 additional studies were excluded, yielding two final studies for consideration.

Two studies ultimately generated direct evidence about the review questions (Islam et al [49] and Wilkinson [50]). Islam et al[49] was published in the peer reviewed literature, while Wilkinson[50] was initially identified in the grey literature, and the full report was provided by the author to the review team.

Included studies

Islam et al

Islam et al was a three-armed randomized controlled trial carried out in Bangladesh in 2012-2015 comparing F-100 (1 kcal/ml, 2.9 g protein/ 100ml; n=50), diluted F-100 (0.77 kcal/ml, 2.2 g protein/100 ml; DF-100, n=52) and standard infant formula (0.70 kcal/ml, 1.6 g protein/100 ml; IF, n=50) in the context of infant rehabilitation from severe acute malnutrition (SAM) (Table 1 below). Islam et. al included infants under 6 months of age admitted for diarrheal illness with weight-for-length z-score of <-3 and/or bipedal edema that were stabilized in a ward for infants requiring long term specialized care less than nine days (free of edema, with cessation of vomiting, fewer than three watery stools /day and no clinical signs of infection). Infants in the three study arms were comparable in most biomedical, socio-economic and demographic characteristics, however, those in the F-100 group were significantly more wasted at the time of enrollment in the trial compared to infants in the other two study arms (p=0.02).

Once stabilized per the above criteria, infants were provided with F-100, DF-100 or standard IF. During the first two days infants received the study diet of 130 ml/kg/day in 12 feeds and then increased by 10 ml per feed until some remained. They were fed every three hours thereafter. Infants were discharged when they gained 15% of their weight or had edema-free weight-for-length z-score ≥ -2. Mothers who were breastfeeding their participant infants were encouraged to continue doing so. Notably, only approximately half of the participants were breastfed on entry into the trial. Additionally, those who were breastfed received a very small proportion of their feeds from breastfeeding (e.g., less than 10% of total kcal/kg/day, on average). Water was also offered between feeds.

If infants became ill during the treatment, they were returned to the ward for infants requiring long term specialized care or another ward for short-term specialized care, fed F-75 (0.75 kcal/ml and 0.9 g protein/100 ml), and returned to the trial once they recovered from illness. A third of participants (n=52; F-100 n=19; DF-100 n= 20; IF n=13) experienced illness (diarrhea and pneumonia) that necessitated transfer to one of the longer term or specialty wards. The number of infants transferred did not statistically differ across the three arms (p=0.29). No information was provided about the length of treatment in the two other wards or additional details of comparability upon returning to the trial.

Wilkinson

Wilkinson's study was a two-armed study comparing DF-100 (0.73 kcal/ml, 1.8 g protein/100 ml, n=72) and a standard infant formula (0.77 kcal/ml, 1.95 g/100 ml; IF, n=74) in the context of a Management of Acute Malnutrition in Infants (MAMI) project (Table 1 below). This study was designed to improve care for acutely malnourished infants under 6 months in Niger and two sites in the Democratic Republic of Congo (DRC) in 2007-2008. It is important to note that data from Niger were ultimately not presented in the final analysis due to small sample size and lack of adherence to the protocol. Due to fewer than expected visits to Therapeutic Feeding Centers (TFCs) during the study period, the study was not adequately powered to detect differences in treatment.

Wilkinson included infants under six months free of any oedema, with maternal report of breastfeeding failure or not gaining weight at home. No blinding was possible due to the appearance and preparation of the supplemental milks. All mothers of participants were breastfeeding or were engaging in relactation and received lactation support; though this was not quantitated in the report. They used the supplementary suckling technique (SST) to stimulate relactation while receiving therapeutic feeding whenever possible. Supplementary feeds were reduced by half when infants were gaining 20 g/day. Finally, supplementation was stopped when infants maintained a weight gain of 10g/day for 3 days in a row. Infants were discharged when they were able to maintain weight gain for 3-5 days. If infants became ill, they were transferred to a hospital and continued to receive supplemental milk. Breastfeeding observations were not continued during hospitalization due to lack of staff. The author's description indicates that the ill infants who were transferred to the hospital remained in the trial although many of whom later died; it is not clear, however, how well protocols were followed for this group. The author notes that pediatric and neonatal services were limited and weak at the hospitals to which ill infants were transferred, with no specialized neonatal care for very young infants.

Attrition, final sample size, and risk of bias

Islam et al experienced high attrition of 24 infants (16%) leaving the trial before discharge criteria were met across all three arms. Attrition did not statistically differ across the three study arms. Supplementary data suggest that 8 additional infants dropped out of the study (see Islam et al Supplementary Table 2). One infant in the F-100 died due to a hospital acquired infection. Consequently, only 36/52 (F-100), 42/52 (DF-100), 42/50 (IF) infants from the three groups, respectively, completed the study to discharge. Islam et al was rated at high risk of bias according to the ROB-2 assessment due to non-adherence to the assigned intervention and loss to follow-up (see Table 1 and Tables 2-4 for details).

The Wilkinson trial also experienced high rates of infants leaving the trial prior to meeting discharge criteria (DF-100 n=13; IF n=10) (no difference between study arms). Additionally, there was a large number of deaths in both arms (DF-100 n=12; IF n=11) (no difference between study arms). One infant from each group was transferred out of the study. A total of 48 infants were recovered in the DF-100 and 50 in the IF group, respectively. These findings did not differ by intervention. Wilkinson's study was rated at high risk of bias according to the ROB-2 assessment due to unblinding and loss to follow-up (see Table 1 and Tables 2-4 for details).

Outcomes of interest

Due to the small number of studies identified by the search, the initial questions for the review could not be fully assessed. We present the outcomes of interest that are available in three GRADE tables (Tables 2-4 below). Table 2 combines the comparison shared by the two included studies (DF-100 compared with IF). Detailed information about the populations and interventions is presented in Table 2 footnotes a) and b) to help contextualize the findings. Additionally, Table 3 presents the F-100 v. IF comparison, and Table 4 presents the F-100 v DF-100 comparison, both from Islam et al.

Rate of weight gain was the only outcome assessed for DF-100 v IF in both studies. Islam et al found significantly higher rates of weight gain for both F-100 (mean difference 4.6; 95% CI 1.5-7.6; p=0.004) and DF-100 (mean difference 3.1; 95% CI 0.6-5.5; p= 0.015) when compared with IF, respectively. In contrast, Wilkinson did not find significant differences in rate of weight gain between DF-100 and IF (0.1 g/kg/day greater for IF-100, 95% CI: -3.3, 3.5).

The weight gain outcome (g/kg/day) was meta-analyzed with the two studies (Islam et al 2019 and Wilkinson 2008) using a random-effects model (Figure 2). The meta-analysis estimate of the mean difference in weight gain between IF and DF-100 was 1.72 g/kg/day, which was not statistically significant (p=0.278); 95% CI: -1.39, 4.83. There was some evidence of moderate heterogeneity across studies, although it was not statistically significant; Q(1)=2.30, p=0.130; I^2=56.5.

Total weight gain at discharge was only reported for Wilkinson and did not significantly differ between the two groups (non-significantly higher weight gain for DF-100 compared with IF; 63.8 g (95% CI: -53.4, 181).

There was one death in Islam's study due to a hospital acquired infection, but it was deemed unrelated to the infant's diet. Mortality was high in both arms of the Wilkinson study (DF-100 n=12; IF n=11) but did not differ by treatment arm. Infants who died were all under 60 days old at admission; 43% (10/23) were less than 30 days old at admission, and 56% (13/23) were less than 60 days old at admission.

Islam et al reported faster recovery for the F-100 group than for the IF group (p=0.043), but the rate of recovery for the F-100 group was not faster than for infants who received DF-100 (p=0.09). There were no differences in rate of recovery or duration of treatment from admission to discharge between the two arms of the Wilkinson study overall. However, there were significant differences between the two centers in duration of treatment, with significantly shorter treatment from admission to discharge for IF compared with DF-100 in Baraka (4.7 days 95% CI: -10.7, 1.3) and significantly longer treatment from admission to discharge for the IF arm compared with DF-100 in Uvira (3.5 days 95% CI: -1.5, 8.5).

Islam et al also examined additional outcomes to assess the safety of F-100 based on previous concerns that it would result in high renal solute load (RSL) and risk of hyponatraemic dehydration. The study did not find evidence of these potential safety issues. The RSL was highest in the F-100 group (126.4 51.9 ± 51.9 mOsm/day) and was significantly different between F-100 and DF-100 as well as between F-100 and IF groups. However, sodium and urinary specific gravity were within normal range (Tables 2-4).

Discussion

The Islam et al trial recommended the use of undiluted F-100 as a simpler approach and did not find any safety concerns in the controlled, hospital environment of the trial. Although it should be noted that none of the infants in the trial had diarrhea, nor had dehydration. This is because in that trial infants who became ill with diarrhea, were temporarily transferred out of the trial and received F-75 coupled with additional treatment. In field practice, however, many growth faltering infants have diarrhea, may be febrile, and/or are fluid restricted for a variety of reasons (e.g. those with cardiac defects, those who are mixed fed and lack access to clean drinking water, etc.). In these situations, the higher solute load of F-100 may pose a risk.

Wilkinson's approach emphasized the use of the supplemental suckling technique (SST) to facilitate relactation and lowered the amount of supplemental milk as infants recovered. However, the trial did not have sufficient resources to record further breastfeeding data or to support those who were transferred to under-resourced hospitals that lacked specialized pediatrics care and skilled lactation support. The study population of Islam et al reflects a very short period of exclusive breastfeeding and any breastfeeding at the time of trial (~50%). Although mothers who were breastfeeding received encouragement to continue and those non-breastfeeding were counseled on relactation, infants only received a very small portion of their energy intake from breastfeeding. Additionally, infants were offered water between feeds, which is not consistent with WHO guidance.

The trials in this review demonstrate the continued need to support lactation in the context of growth faltering as part of an integrated approach protecting, promoting, and supporting breastfeeding. Although WHO guidance is clear that exclusive breastfeeding for 6 months

ensures optimal infant feeding and development, to date, the focus of growth faltering interventions has been on the supplemental milks that may be used in these contexts without paying enough attention to supporting breastfeeding or relactation. A recent observational study[51] of LBW infants in resource limited settings similarly identified a need for “universal and consistent” lactation support. Moreover, a recent systematic review[52] identified additional need for high quality studies of interventions to address breastfeeding difficulties among mothers of infants under 6 months with growth faltering. Our review identified a study protocol that addresses growth faltering in infants under 6 months in Asia and Africa in the context of fully implementing WHO Breastfeeding Counselling Guidelines on breastfeeding promotion and support (PI: Bhandari).[53] This study has not started recruitment.

~~Conclusion~~

Overall, t~~T~~here is very limited high-quality evidence on interventions to understand the nutritional management of infants under 6 months experiencing growth faltering. Of the two trials identified by this search, only one has been published in the peer reviewed literature. Both trials had small sample sizes, and high numbers of participants leaving the trial before discharge criteria were met. This limits the generalizability of the findings. In Islam’s trial the authors noted that the F-100 study arm differed between the other two study arms in greater wasting at enrollment. Additionally, over a third of infants temporarily paused their participation in the trial while transferred out due to illness, and then were later returned to the trial, which poses methodological challenges for comparability. For Wilkinson’s trial the inability to adequately power the trial and the lack of blinding posed challenges. There were additional non-statistically significant differences in the findings between the two centers, with longer duration of treatment from admission to discharge for the diluted F-100 group at Baraka versus longer duration for the IF group at Uvira. Finally, the design of the two studies differed substantially, and only one outcome (rate of weight gain) was shared among them for the diluted F-100 and IF comparison. The lack of studies combined with the low quality of the only two studies available is quite concerning given the strong need for evidence-based guidelines for the nutritional management of young malnourished children. This gap has been identified for decades. It is indeed striking that many national guidelines on the treatment of acutely malnourished children include F-75, diluted F-100 or infant formula for infants under 6 months in the recommendations without having the level of evidence needed to support this.

Another key concern in this area is the lack of a well-thought-out framework that can help improve the design for studies in this area, which accounts for the need to first and foremost protect breastfeeding among malnourished infants. A current Cochrane review is examining whether lower protein content formulas can address malnutrition without the adverse longer-term outcomes of obesity and metabolic disorders[54]. High income settings face additional considerations on the use of fortifiers and modular nutrients for preterm infants, including cost and cross-contamination of using pumps to express milk to nutrient enrich a supply.[55]

We specifically propose a new research framework that is consistent with the MAMI pathway[56] package’s emphasis on continuing/restoring breastfeeding whenever possible, and supports continuation of breastfeeding, relactation counseling, and expands this framework with the use of human milk. We recommend conducting a series of linked studies to inform an evidence-based decision tree to understand if, when and for how long the malnourished child

needs to be supplemented with supplemental milks and which kind of supplemental milks are needed (Figure 3). In full consistency with our systematic review's conclusion, the revised 2023 WHO guideline on the prevention and management of wasting and nutritional oedema (acute malnutrition) in infants and children under 5 years also emphasizes the importance of supporting and restoring optimal breastfeeding practices to prevent malnutrition and in the context of continued support for recovery from acute malnutrition[57]. In caring for hospitalized infants that are at elevated risk of growth faltering (e.g. very low birth weight infant or infants with cardiac defects), there has been a paradigm shift in the past 30 years from the almost exclusive use of nutrient-enriched specialty formulas to a variety of strategies to preserve human milk feeding, but without rigorously designed research studies. These strategies have included breastfeeding support, increasing the volumes of breast milk fed, and adding nutrient fortifiers or less expensive nutrient modulars to expressed human milk (mother's own or donor human milk) fed by feeding bottle, tube or a supplemental nursing system. Recent WHO guidance on caring for preterm and LBW infants states that mother's own milk (MOM) is recommended for this group, and there is limited to no benefits to using IF in comparison to MOM[6]. The evidence base for the guidance suggests that breastfeeding is likely an undervalued intervention for these vulnerable infants. Although this guidance is not specifically directed at infants with growth faltering, it has implications for interventions to address growth faltering in settings with high prevalence of malnutrition. Given that practitioners need to make feeding decisions with very little evidence to guide them, it is important to also learn from successful practical experiences managing the nutritional needs of infants who are not growing well across diverse settings, and carefully evaluate them.

The trials also call attention to the importance of translation of research through implementation of interventions that is responsive to the needs of diverse local contexts with a high prevalence of growth faltering. A key issue for both trials in our analysis was that there were infants leaving the trial before discharge criteria were met. When caregivers have competing demands on their time, they may struggle to keep their infants in treatment, and sustaining recovery may also be a challenge. They may have labor and other caregiving demands that prevent them from being able to stay at a hospital or recovery center. To ensure recovery and sustained growth and healthy development, a broader evidence-informed policy approach is necessary to ensure support for families facing these challenges.

Conclusion and r~~R~~esearch recommendations

Critical questions remain about how to best prevent, and support recovery, from growth faltering in the context of malnutrition. The two studies we identified in this systematic review provide limited generalizability. The inequities in the prevalence of growth faltering under 6 months between those in low- and middle-income settings compared with high-income settings point to the importance of addressing the broader social inequities that are the upstream drivers of malnutrition.[58] Additional research needs to address the needs of infants at risk for growth faltering and those facing complex medical conditions. We recommend a paradigm shift for this research that reorients breastfeeding and the use of human milk and skin to skin contact as foundational even to medically fragile infants. In this paradigm, the focus in malnutrition research would be on supporting and restoring breastfeeding, rather than previous approaches that focus research questions on supplemental milk products. A similar paradigm shift has

already taken place in preterm infant feeding research and practice, as reflected by recent WHO guidance[6]. Further investment is necessary to ensure that the protection, promotion and support of breastfeeding is at the center of this work, even in complex humanitarian emergencies.

Acknowledgements

The authors would like to thank the Johns Hopkins University Welch Library team for their assistance.

Data availability statement

All data are publicly available. The search strategy as well as the included and excluded studies can be found in the manuscript.

Author contributions

CT, DOC, MV, and RPE conceptualized the study. DOA and KP carried out the review and analyses of findings, supported by the entire author team. CB provided additional analyses. All authors contributed to the development of the final manuscript.

Competing interests statement

The authors declare no competing interests.

Funding

This study was funded by the World Health Organization (WHO). WHO commissioned the review and as such provided guidance on the development of the questions examined by the review, and reviewed and provided methodological feedback on the technical report. WHO did not review or provided feedback on the interpretation of findings or recommendations from this article. These were made by the article's authors and do not necessarily represent the views from the WHO.

Captions

Figure 1. PRISMA flow chart

Figure 2. Meta-analysis of weight gain

Figure 3. Research framework to guide the design of future studies to inform comprehensive evidence-based guidelines for recovery from growth faltering in infants <6 months. Note: Supplemental milk is defined as donor human milk, fortified donor human milk, fortified mothers' own milk, human milk from wet nurse, commercial infant formula, nutrient-enriched commercial infant formula, F-75, F-100, or diluted F-100.

Table 1. Summary of included studies (Islam et al 2019, Wilkinson 2008).

Table 2. GRADE Evidence Profile: Diluted F-100 compared to infant formula for infants <6 months of age with severe wasting and/or oedema Author(s): Tomori et al.

Question: Diluted F-100 compared to infant formula for infants <6 months with severe wasting and/or oedema.

Table 3. GRADE Evidence Profile: F-100 compared to infant formula for infants <6 months of age with severe wasting and/or oedema Author(s): Tomori et al.
Question: F-100 compared to infant formula for infants <6 months with severe wasting and/or oedema.

Table 4. GRADE Evidence Profile: F-100 compared to diluted F-100 for infants <6 months of age with severe wasting and/or oedema Author(s): Tomori et al.
Question: F-100 compared to diluted F-100 for infants <6 months with severe wasting and/or oedema

References

1. Victora CG, De Onis M, Hallal PC, Blössner M, Shrimpton R. Worldwide timing of growth faltering: revisiting implications for interventions. *Pediatrics* 2010; **125**(3): e473-e80.
2. Rana R, McGrath M, Gupta P, Thakur E, Kerac M. Feeding Interventions for Infants with Growth Failure in the First Six Months of Life: A Systematic Review. *Nutrients* 2020; **12**(7): 2044.
3. Kerac M, Blencowe H, Grijalva-Eternod C, et al. Prevalence of wasting among under 6-month-old infants in developing countries and implications of new case definitions using WHO growth standards: a secondary data analysis. *Archives of disease in childhood* 2011; **96**(11): 1008-13.
4. UNICEF/WHO/World Bank Group. Levels and trends in child malnutrition. Geneva, Switzerland: UNICEF, WHO, World Bank Group, 2021.
5. National Institute for Health Care Excellence. Faltering growth: recognition and management of faltering growth in children. NICE London (United Kingdom); 2017.
6. World Health Organization. WHO recommendations for care of the preterm or low-birth-weight infant. Geneva, Switzerland: World Health Organization, 2022.
7. Benjamin-Chung J, Mertens A, Colford JM, et al. Early childhood linear growth faltering in low- and middle-income countries. *medRxiv* 2021: 2020.06.09.20127001.
8. Lelijveld N, Seal A, Wells JC, et al. Chronic disease outcomes after severe acute malnutrition in Malawian children (ChroSAM): a cohort study. *The Lancet Global Health* 2016; **4**(9): e654-e62.
9. Grey K, Gonzales GB, Abera M, et al. Severe malnutrition or famine exposure in childhood and cardiometabolic non-communicable disease later in life: a systematic review. *BMJ global health* 2021; **6**(3): e003161.
10. Grijalva- Eternod CS, Kerac M, McGrath M, et al. Admission profile and discharge outcomes for infants aged less than 6 months admitted to inpatient therapeutic care in 10 countries. A secondary data analysis. *Maternal & child nutrition* 2017; **13**(3): e12345.
11. Campion-Smith TJ, Kerac M, McGrath M, Berkley JA. Antimicrobial and micronutrient interventions for the management of infants under 6 months of age identified with severe malnutrition: a literature review. *PeerJ* 2020; **8**: e9175.

12. Black RE, Victora CG, Walker SP, et al. Maternal and child undernutrition and overweight in low-income and middle-income countries. *Lancet* 2013; **382**(9890): 427-51.
13. Han Z, Lutsiv O, Mulla S, McDonald SD. Maternal height and the risk of preterm birth and low birth weight: a systematic review and meta-analyses. *J Obstet Gynaecol Can* 2012; **34**(8): 721-46.
14. Khatun W, Rasheed S, Alam A, Huda TM, Dibley MJ. Assessing the intergenerational linkage between short maternal stature and under-five stunting and wasting in Bangladesh. *Nutrients* 2019; **11**(8): 1818.
15. Rijken MJ, De Livera AM, Lee SJ, et al. Quantifying low birth weight, preterm birth and small-for-gestational-age effects of malaria in pregnancy: a population cohort study. *PLoS One* 2014; **9**(7): e100247.
16. Saito M, Briand V, Min AM, McGready R. Deleterious effects of malaria in pregnancy on the developing fetus: a review on prevention and treatment with antimalarial drugs. *The Lancet Child & Adolescent Health* 2020; **4**(10): 761-74.
17. Abdi F, Alimoradi Z, Alidost F. Pregnancy outcomes and effects of antiretroviral drugs in HIV-positive pregnant women: a systematic review. *Future Virology* 2019; **14**(3): 197-210.
18. Ko JY, Desisto CL, Simeone RM, et al. Adverse Pregnancy Outcomes, Maternal Complications, and Severe Illness Among US Delivery Hospitalizations With and Without a Coronavirus Disease 2019 (COVID-19) Diagnosis. *Clinical Infectious Diseases* 2021; **73**(Supplement_1): S24-S31.
19. Husseini M, Darboe MK, Moore SE, Nabwera HM, Prentice AM. Thresholds of socio-economic and environmental conditions necessary to escape from childhood malnutrition: a natural experiment in rural Gambia. *BMC Medicine* 2018; **16**(1).
20. Brander RL, Pavlinac PB, Walson JL, et al. Determinants of linear growth faltering among children with moderate-to-severe diarrhea in the Global Enteric Multicenter Study. *BMC medicine* 2019; **17**: 1-16.
21. Ross KM, Guardino C, Dunkel Schetter C, Hobel CJ. Interactions between race/ethnicity, poverty status, and pregnancy cardio-metabolic diseases in prediction of postpartum cardio-metabolic health. *Ethnicity & health* 2020; **25**(8): 1145-60.
22. Li F, Wang T, Chen L, Zhang S, Chen L, Qin J. Adverse pregnancy outcomes among mothers with hypertensive disorders in pregnancy: a meta-analysis of cohort studies. *Pregnancy Hypertension* 2021.
23. Relph S, Patel T, Delaney L, Sobhy S, Thangaratinam S. Adverse pregnancy outcomes in women with diabetes-related microvascular disease and risks of disease progression in pregnancy: A systematic review and meta-analysis. *PLOS Medicine* 2021; **18**(11): e1003856.
24. Kaldenbach S, Engebretsen IM, Haskins L, Conolly C, Horwood C. Infant feeding, growth monitoring and the double burden of malnutrition among children aged 6 months and their mothers in KwaZulu- Natal, South Africa. *Maternal & Child Nutrition* 2022; **18**(1): e13288.
25. Pérez-Escamilla R. Food security and the 2015–2030 sustainable development goals: From human to planetary health: Perspectives and opinions. *Current developments in nutrition* 2017; **1**(7): e000513.
26. Fleischer NL, Merialdi M, van Donkelaar A, et al. Outdoor air pollution, preterm birth, and low birth weight: analysis of the world health organization global survey on maternal and perinatal health. *Environmental health perspectives* 2014; **122**(4): 425-30.

27. Yuan L, Zhang Y, Gao Y, Tian Y. Maternal fine particulate matter (PM2.5) exposure and adverse birth outcomes: an updated systematic review based on cohort studies. *Environmental Science and Pollution Research* 2019; **26**(14): 13963-83.
28. Tusting LS, Bradley J, Bhatt S, et al. Environmental temperature and growth faltering in African children: a cross-sectional study. *The Lancet Planetary Health* 2020; **4**(3): e116-e23.
29. Dadi AF, Miller ER, Mwanri L. Postnatal depression and its association with adverse infant health outcomes in low- and middle-income countries: a systematic review and meta-analysis. *BMC Pregnancy Childbirth* 2020; **20**(1): 416.
30. Surkan PJ, Patel SA, Rahman A. Preventing infant and child morbidity and mortality due to maternal depression. *Best Pract Res Clin Obstet Gynaecol* 2016; **36**: 156-68.
31. Surkan PJ, Kennedy CE, Hurley KM, Black MM. Maternal depression and early childhood growth in developing countries: systematic review and meta-analysis. *Bull World Health Organ* 2011; **89**(8): 608-15.
32. Lawrence RM, Lawrence RA. Normal Growth, Growth Faltering, and Obesity in Breastfed Infants. Breastfeeding: Elsevier; 2022: 298-325.
33. Ghanchi A, Derridj N, Bonnet D, Bertille N, Salomon LJ, Khoshnood B. Children Born with Congenital Heart Defects and Growth Restriction at Birth: A Systematic Review and Meta-Analysis. *Int J Environ Res Public Health* 2020; **17**(9).
34. Mekonnen AG, Odo DB, Nigatu D, Sav A, Abagero KK. Women’s empowerment and child growth faltering in Ethiopia: evidence from the Demographic and Health Survey. *BMC Womens Health* 2021; **21**(1).
35. Na M, Jennings L, Talegawkar SA, Ahmed S. Association between women’s empowerment and infant and child feeding practices in sub-Saharan Africa: an analysis of Demographic and Health Surveys. *Public health nutrition* 2015; **18**(17): 3155-65.
36. von Salmuth V, Brennan E, Kerac M, McGrath M, Frison S, Lelijveld N. Maternal-focused interventions to improve infant growth and nutritional status in low-middle income countries: A systematic review of reviews. *PLoS One* 2021; **16**(8): e0256188.
37. Pérez-Escamilla R, Hromi-Fiedler A, Rhodes E, et al. Impact of prelacteal feeds and neonatal introduction of breastmilk substitutes on breastfeeding outcomes: A systematic review and meta-analysis. *Matern Child Nutr* 2022; **18**: e13368.
38. Vilar- Compte M, Pérez- Escamilla R, Orta- Aleman D, et al. Impact of baby behaviour on caregiver's infant feeding decisions during the first 6 months of life: A systematic review. *Maternal & Child Nutrition* 2022; **18**(S3): e13345.
39. Martin A, Connelly A, Bland RM, Reilly JJ. Health impact of catch-up growth in low-birth weight infants: systematic review, evidence appraisal, and meta-analysis. *Matern Child Nutr* 2017; **13**(1).
40. Amugsi DA, Dimbuene ZT. Basic determinants of child linear growth outcomes in sub-Saharan Africa: a cross-sectional survey analysis of positive deviants in poor households. *Scientific Reports* 2022; **12**(1): 14218.
41. Organization. WH. WHO recommendations for care of the preterm or low-birth-weight infant. Geneva, Switzerland: World Health Organization, 2022.
42. DiLauro S, Unger S, Stone D, O'Connor DL. Human milk for ill and medically compromised infants: strategies and ongoing innovation. *Journal of Parenteral and Enteral Nutrition* 2016; **40**(6): 768-82.
43. Aneja S, Kumar P, Choudhary TS, et al. Growth faltering in early infancy: highlights from a two-day scientific consultation. BMC proceedings; 2020: Springer; 2020. p. 1-7.

44. Sarkis-Onofre R, Catalá-López F, Aromataris E, Lockwood C. How to properly use the PRISMA Statement. *Systematic Reviews* 2021; **10**(1): 1-3.
45. Sterne J, Savović J, Page M, et al. RoB 2: a revised tool for assessing risk of bias in randomised trials. . *BMJ* 2019; **366**: l4898.
46. Alonso-Coello P, Schünemann HJ, Moberg J, et al. GRADE Evidence to Decision (EtD) frameworks: a systematic and transparent approach to making well informed healthcare choices. 1: Introduction. *bmj* 2016; **353**.
47. Clarke SE, Evans S, Macdonald A, Davies P, Booth IW. Randomized comparison of a nutrient-dense formula with an energy-supplemented formula for infants with faltering growth. *J Hum Nutr Diet* 2007; **20**(4): 329-39.
48. Evans S, Twaissi H, Daly A, Davies P, Macdonald A. Should high-energy infant formula be given at full strength from its first day of usage? *J Hum Nutr Diet* 2006; **19**(3): 191-7; quiz 9.
49. Islam MM, Huq S, Hossain MI, et al. Efficacy of F-100, diluted F-100, and infant formula as rehabilitation diet for infants aged < 6 months with severe acute malnutrition: a randomized clinical trial. *Eur J Nutr* 2020; **59**(5): 2183-93.
50. Wilkinson C. Comparative study of the effectiness of infant formula and diluted F-100 therapeutic milk products in the treatment of severe acute malnutrition in infants under six months of age: Technical Department Research Service ACF-France, 2008.
51. Vesel L, Bellad RM, Manji K, et al. Feeding practices and growth patterns of moderately low birthweight infants in resource-limited settings: results from a multisite, longitudinal observational study. *BMJ open* 2023; **13**(2): e067316.
52. Mohandas S, Rana R, Sirwani B, Kirubakaran R, Puthussery S. Effectiveness of Interventions to Manage Difficulties with Breastfeeding for Mothers of Infants under Six Months with Growth Faltering: A Systematic Review Update. *Nutrients* 2023; **15**(4): 988.
53. Clinical Trials Registry - India CTRI/2022/07/043716. Nutritional management of growth faltering in infants aged under six months in Asia and Africa. 2022. http://www.ctri.nic.in/Clinicaltrials/pmaindet2.php?trialid=70345 (accessed November 19 2022).
54. Gonzalez Garay AG, Medina Vera I, Serralde-Zúñiga AE, Velasco Hidalgo L, Alonso Ocaña MV. Higher versus lower protein intake in formula- fed term infants. *Cochrane Database of Systematic Reviews* 2020; (10).
55. Pereira-Da-Silva L, Virella D, Fusch C. Nutritional Assessment in Preterm Infants: A Practical Approach in the NICU. *Nutrients* 2019; **11**(9): 1999.
56. Grey K, Brennan E, Kerac M, McGrath M. The MAMI Care Pathway Package: A resource to support the management of small and nutritionally at-risk infants under six months of age and their mothers (MAMI). *South Sudan Medical Journal* 2021; **14**(3): 94-7.
57. World Health Organization. WHO guideline on the prevention and management of wasting and nutritional oedema (acute malnutrition) in infants and children under 5 years Geneva, Switzerland: World Health Organization, 2023.
58. Pérez-Escamilla R, Bermudez O, Buccini GS, et al. Nutrition disparities and the global burden of malnutrition. *BMJ* 2018; **361**: k2252.

We appreciate the opportunity to revise our manuscript and respond to the reviewers' concerns. Please see our point by point revisions below.

Reviewer #1: 1. Please define some of the statistical symbols for the reader.
We thank the reviewer and have defined these symbols in the text.

2.Indicate the period of search? Is it from 1980 to when?
It is from 1980 to 2022. We have revised the text accordingly.

3. The final selection of 2 studies under systematic review:
This looks too few to make conclusive recommendations. Is it possible to expand the selection criteria? It will be nice to review studies from more developed, developing (Bangladesh not sufficient) and even Sub-Saharan Africa (DRC, Niger: not sufficient)!. Should we assume that there are only 2 studies relevant for the study? What was the main limitations in arriving at a substantial number of studies?

We thank the reviewer for these comments. The World Health Organization guideline process, for which this review was commissioned, required that we only include studies that met very specific, and narrow inclusion criteria. Accordingly, only randomized controlled trials, cluster-randomized trials, and quasi-experimental studies/non-randomized controlled studies were included. Qualitative studies, reviews, and systematic reviews, as well as observational studies were excluded. This is the reason why we were unable to include the additional studies suggested by the reviewer.

After our very extensive and extremely thorough search process that followed NASEM guidelines for a systematic review, we were only able to identify two relevant studies, as we state in the text. As indicated in the discussion (lines 429-448) this was indeed an important finding highlighting the need for supporting much more and better research in this field to inform evidence-based guidance moving forward.

We incorporated the suggested references as background, but we are not able to expand the search as the criteria were specified by the WHO and we registered our protocol in advance of carrying out the study. As mentioned above, we made recommendations for future research so that guidelines in the future can be informed by a richer body of evidence.

4. Demographic and Health Survey (DHS) are conducted in almost all regions of the world and the data is regularly available. It has a module on infant feeding and nutrition outcomes including growth faltering. Please include some findings and how they relate with current study.

We incorporated the suggested literature into the contextualization of our study (see especially lines 78-87 and lines 117-124). We also discussed the specific requirements of this systematic review undertaken for the WHO guideline development process (lines 133-139 and 143-162) and discussed the limited evidence on the specific PICO question we were asked to answer in the Discussion (see lines 429-448).

5.There are recent studies on growth faltering. Consider reviewing many of these studies and what extent do these inform your study?

We have reviewed the relevant literature and included it as context as stated above (see response to Question 4) and in the references in the Introduction, which include recent systematic reviews on interventions addressing growth faltering.

Reviewer #2: This article is well written, I have only two recommendations:

We thank the reviewer for these comments.

1. Some of the content of conclusion can be better placed in discussion section.

We have moved some of the text from the Conclusion into the Discussion.

2. All journal titles should be abbreviated as per the 'NLM Catalog of Journals'.

We have revised journal abbreviations according to the NLM Catalog.

Reviewer #3: This is a well-written manuscript that only needs to undergo a few minor changes. It has clear research questions, good flow of search strategy ,and supported with evidence on excluded studies with justification. It is technically sound. Though the study has heterogeneity, the magnitude is also estimated statistically. I really enjoyed reviewing.

We thank the reviewer for these comments.

Additional responses to the attached peer review report

Peer Review Report

Manuscript Title: Critical Research Gaps in Treating Growth Faltering in Infants under 6 Months: A Systematic Review and Meta-Analysis

1. Summary of the research and your overall impression

The systematic review and meta-analysis aimed at identifying/evaluating the best method for intervening in infants (< 6 months) suffering from growth failure. It focused on supplementary feeding with therapeutic feedings, mother milk, infant formula, and the importance of breastfeeding support. Given limited evidence on the subject of interest, the study tried to review a very small number of pieces of literature that met the inclusion criteria. The electronic search strategy was applied with two reviewers who were blinded to the authors. Two randomized

controlled trials were used in the analysis, which were different in enrollment criteria in terms of population characteristics and methodology followed. This had made the combined estimate difficult for the outcome of interest, except for weight gain.

The manuscript's strengths are:

- Problem is stated in a clear way
- Clear research questions
- Good flow of search strategy and supported with evidence on excluded studies with justification
- Quality of score of each study assessed
- Though the study has heterogeneity, the magnitude is estimated by the I^2 statistic
- Since the finding did not fully address the intended objective, further research is also recommended

We thank the reviewer for highlighting the strengths of the manuscript.

Weaknesses:

- Flow of ideas sometimes interrupted, re-explained in different subsections

We have revised the manuscript to improve flow.

- Unclear whether publication bias is assessed

In accordance with recommendations by the Cochrane Handbook for Systematic Reviews we could not assess publication bias due to the small set of included studies (guidance recommends a minimum of 10 studies for this assessment).

- Topic excluded in the study (pre-term infants) stated in the discussion part which is not supported by the result

The discussion of preterm infants in the discussion is highly relevant for the broader approach to the framework for prevention of malnutrition that we propose in the paper. Please note that statements are in relationship to the recent WHO guidance focused on nutrition recommendations for premature babies. That guidance was issued by a separate expert committee than the one that commissioned our review so it was very important to link both in the discussion.

2. Discussion of specific areas for improvement

Major issues:

- Unclear whether publication bias is assessed

Per Cochrane Handbook for Systematic Reviews publication bias was not assessed due to the small set of included studies (guidance recommends a minimum of 10 studies for this assessment).

The authors stated ‘’risk of bias was assessed for meta-analyses’’ on line 180. Thus, the authors should include more information that clarifies this statement

We deleted this sentence for clarity since we assessed risk of bias for the studies but did not assess publication bias per Cochrane Handbook guidance as mentioned above.

Minor issues:

- Lines 450-456; It has appeared in the conclusions part, there is a statement that is out of the result found.

As mentioned in response above related to same issue, we refer to the preterm guidance because it is helpful to consider it in the broader context of guidance aiming to achieve optimal nutrition and prevent malnutrition. We specify these implications in the text (lines 480-485) as follows:

"Although this guidance is not specifically directed at infants with growth faltering, it has implications for interventions to address growth faltering in settings with high prevalence of malnutrition. Given that that practitioners have had the need to make feeding recommendations with very little evidence to guide them, it is important to also learn from the recent WHO guidance for premature babies and successful practical experiences managing the nutritional needs of infants who are not growing well across diverse settings, and carefully evaluate them. "

- Checklist (PRISMA) is filled, but not location-specific (row number, line number missing)

We have revised the PRISMA checklist to include line numbers.

- Lengthy explanations under Table 2-4.

The explanations follow the protocol for the evidence tables per the Grading of Recommendations, Assessment, Development and Evaluations (GRADE) methodology and the GRADE Pro software. This level of detail is also expected for a review undertaken for the WHO's guideline process.
