# Supplementary material for: Critical research gaps in treating growth faltering in infants under 6 months: A systematic review and meta-analysis
Source: PLOS Glob Public Health. 2024 Jan 8;4(1):e0001860. doi: 10.1371/journal.pgph.0001860 (PMC10773941; doi:10.1371/journal.pgph.0001860)
Supplement: Attachment — Submitted filename: Peer Review Report.docx [file pgph.0001860.s005.docx]

Peer Review Report

Manuscript Title: Critical Research Gaps in Treating Growth Faltering in Infants under 6 Months: A Systematic Review and Meta-Analysis

### **1. Summary of the research and your overall impression**

The systematic review and meta-analysis aimed at identifying/evaluating the best method for intervening in infants (< 6 months) suffering from growth failure. It focused on supplementary feeding with therapeutic feedings, mother milk, infant formula, and the importance of breastfeeding support. Given limited evidence on the subject of interest, the study tried to review a very small number of pieces of literature that met the inclusion criteria. The electronic search strategy was applied with two reviewers who were blinded to the authors. Two randomized controlled trials were used in the analysis, which were different in enrollment criteria in terms of population characteristics and methodology followed. This had made the combined estimate difficult for the outcome of interest, except for weight gain.

The manuscript’s strengths are:

- Problem is stated in a clear way
- Clear research questions
- Good flow of search strategy and supported with evidence on excluded studies with justification
- Quality of score of each study assessed
- Though the study has heterogeneity, the magnitude is estimated by the I^2^ statistic
- Since the finding did not fully address the intended objective, further research is also recommended

Weaknesses:

- Flow of ideas sometimes interrupted, re-explained in different subsections
- Unclear whether publication bias is assessed
- Topic excluded in the study (pre-term infants) stated in the discussion part which is not supported by the result

### **2. Discussion of specific areas for improvement**

Major issues:

- Unclear whether publication bias is assessed

The authors stated ‘’risk of bias was assessed for meta-analyses’’ on line 180. Thus, the authors should include more information that clarifies this statement

Minor issues:

- Lines 450-456; It has appeared in the conclusions part, there is a statement that is out of the result found.
- Checklist (PRISMA) is filled, but not location-specific (row number, line number missing)
- Lengthy explanations under Table 2-4.
