# Supplementary material for: Critical research gaps in treating growth faltering in infants under 6 months: A systematic review and meta-analysis
Source: PLOS Glob Public Health. 2024 Jan 8;4(1):e0001860. doi: 10.1371/journal.pgph.0001860 (PMC10773941; doi:10.1371/journal.pgph.0001860)
Supplement: Attachment — Submitted filename: Response to reviewers 8-5-23.docx [file pgph.0001860.s006.docx]

We appreciate the opportunity to revise our manuscript and respond to the reviewers’ concerns. Please see our point by point revisions below.

Reviewer #1: 1. Please define some of the statistical symbols for the reader.

We thank the reviewer and have defined these symbols in the text.

2.Indicate the period of search? Is it from 1980 to when?

It is from 1980 to 2022. We have revised the text accordingly.

3. The final selection of 2 studies under systematic review:
This looks too few to make conclusive recommendations. Is it possible to expand the selection criteria? It will be nice to review studies from more developed, developing (Bangladesh not sufficient) and even Sub-Saharan Africa (DRC, Niger: not sufficient)!. Should we assume that there are only 2 studies relevant for the study? What was the main limitations in arriving at a substantial number of studies?

We thank the reviewer for these comments. The World Health Organization guideline process, for which this review was commissioned, required that we only include studies that met very specific, and narrow inclusion criteria. Accordingly, only randomized controlled trials, cluster-randomized trials, and quasi-experimental studies/non-randomized controlled studies were included. Qualitative studies, reviews, and systematic reviews, as well as observational studies were excluded. This is the reason why we were unable to include the additional studies suggested by the reviewer.

After our very extensive and extremely thorough search process that followed NASEM guidelines for a systematic review, we were only able to identify two relevant studies, as we state in the text. As indicated in the discussion (lines 429-448) this was indeed an important finding highlighting the need for supporting much more and better research in this field to inform evidence-based guidance moving forward.

We incorporated the suggested references as background, but we are not able to expand the search as the criteria were specified by the WHO and we registered our protocol in advance of carrying out the study. As mentioned above, we made recommendations for future research so that guidelines in the future can be informed by a richer body of evidence.

4. Demographic and Health Survey (DHS) are conducted in almost all regions of the world and the data is regularly available. It has a module on infant feeding and nutrition outcomes including growth faltering. Please include some findings and how they relate with current study.

We incorporated the suggested literature into the contextualization of our study (see especially lines 78-87 and lines 117-124). We also discussed the specific requirements of this systematic review undertaken for the WHO guideline development process (lines 133-139 and 143-162) and discussed the limited evidence on the specific PICO question we were asked to answer in the Discussion (see lines 429-448).

5.There are recent studies on growth faltering. Consider reviewing many of these studies and what extent do these inform your study?

We have reviewed the relevant literature and included it as context as stated above (see response to Question 4) and in the references in the Introduction, which include recent systematic reviews on interventions addressing growth faltering.

Reviewer #2: This article is well written, I have only two recommendations:

We thank the reviewer for these comments.

1. Some of the content of conclusion can be better placed in discussion section.

We have moved some of the text from the Conclusion into the Discussion.

2. All journal titles should be abbreviated as per the ‘NLM Catalog of Journals’.

We have revised journal abbreviations according to the NLM Catalog.

Reviewer #3: This is a well-written manuscript that only needs to undergo a few minor changes. It has clear research questions, good flow of search strategy ,and supported with evidence on excluded studies with justification. It is technically sound. Though the study has heterogeneity, the magnitude is also estimated statistically. I really enjoyed reviewing.

We thank the reviewer for these comments.

**Additional responses to the attached peer review report**

Peer Review Report

Manuscript Title: Critical Research Gaps in Treating Growth Faltering in Infants under 6 Months: A Systematic Review and Meta-Analysis

### **1. Summary of the research and your overall impression**

The systematic review and meta-analysis aimed at identifying/evaluating the best method for intervening in infants (< 6 months) suffering from growth failure. It focused on supplementary feeding with therapeutic feedings, mother milk, infant formula, and the importance of breastfeeding support. Given limited evidence on the subject of interest, the study tried to review a very small number of pieces of literature that met the inclusion criteria. The electronic search strategy was applied with two reviewers who were blinded to the authors. Two randomized controlled trials were used in the analysis, which were different in enrollment criteria in terms of population characteristics and methodology followed. This had made the combined estimate difficult for the outcome of interest, except for weight gain.

The manuscript’s strengths are:

- Problem is stated in a clear way
- Clear research questions
- Good flow of search strategy and supported with evidence on excluded studies with justification
- Quality of score of each study assessed
- Though the study has heterogeneity, the magnitude is estimated by the I^2^ statistic
- Since the finding did not fully address the intended objective, further research is also recommended

We thank the reviewer for highlighting the strengths of the manuscript.

Weaknesses:

- Flow of ideas sometimes interrupted, re-explained in different subsections

We have revised the manuscript to improve flow.

- Unclear whether publication bias is assessed

In accordance with recommendations by the Cochrane Handbook for Systematic Reviews we could not assess publication bias due to the small set of included studies (guidance recommends a minimum of 10 studies for this assessment).

- Topic excluded in the study (pre-term infants) stated in the discussion part which is not supported by the result

The discussion of preterm infants in the discussion is highly relevant for the broader approach to the framework for prevention of malnutrition that we propose in the paper. Please note that statements are in relationship to the recent WHO guidance focused on nutrition recommendations for premature babies. That guidance was issued by a separate expert committee than the one that commissioned our review so it was very important to link both in the discussion.

### **2. Discussion of specific areas for improvement**

Major issues:

- Unclear whether publication bias is assessed

Per Cochrane Handbook for Systematic Reviews publication bias was not assessed due to the small set of included studies (guidance recommends a minimum of 10 studies for this assessment).

The authors stated ‘’risk of bias was assessed for meta-analyses’’ on line 180. Thus, the authors should include more information that clarifies this statement

We deleted this sentence for clarity since we assessed risk of bias for the studies but did not assess publication bias per Cochrane Handbook guidance as mentioned above.

Minor issues:

- Lines 450-456; It has appeared in the conclusions part, there is a statement that is out of the result found.

As mentioned in response above related to same issue, we refer to the preterm guidance because it is helpful to consider it in the broader context of guidance aiming to achieve optimal nutrition and prevent malnutrition. We specify these implications in the text (lines 480-485) as follows:

“Although this guidance is not specifically directed at infants with growth faltering, it has implications for interventions to address growth faltering in settings with high prevalence of malnutrition. Given that that practitioners have had the need to make feeding recommendations with very little evidence to guide them, it is important to also learn from the recent WHO guidance for premature babies and successful practical experiences managing the nutritional needs of infants who are not growing well across diverse settings, and carefully evaluate them. “

- Checklist (PRISMA) is filled, but not location-specific (row number, line number missing)

We have revised the PRISMA checklist to include line numbers.

- Lengthy explanations under Table 2-4.

The explanations follow the protocol for the evidence tables per the Grading of Recommendations, Assessment, Development and Evaluations (GRADE) methodology and the GRADE Pro software. This level of detail is also expected for a review undertaken for the WHO’s guideline process.
