# Supplementary material for: Critical research gaps in treating growth faltering in infants under 6 months: A systematic review and meta-analysis
Source: PLOS Glob Public Health. 2024 Jan 8;4(1):e0001860. doi: 10.1371/journal.pgph.0001860 (PMC10773941; doi:10.1371/journal.pgph.0001860)
Supplement: Attachment — Submitted filename: Response to reviewers PGPH 10-9-23.docx [file pgph.0001860.s008.docx]

Response to reviewers PGPH-D-23-00601R1

We thank the reviewers for their comments. We appreciate Reviewer 3’s kind comments and glad that they are satisfied with our revision. We respond to Reviewer 1’s additional comments below.

**Comments to the Author**

**Reviewer #1**: 1. Is this section of included studies part of the study findings or it is supposed to be under methodology? There is need for consistent flow.

We respectfully disagree with the Reviewer. We have followed NASEM recommendations for systematic reviews throughout our paper. Here, we are discussing the characteristics of the studies that have been identified by the systematic review. According to NASEM and other expert systematic reviews bodies on the standard way of reporting systematic reviews, such as PRISMA, this text is part of our findings and therefore belongs in the Results section. Likewise, and as recommended, our results section also includes the discussion of the identified studies’ methodology, which is a component of the review’s findings that is essential to include in this section prior to interpreting the studies’ findings. This manuscript structure that we followed is considered to be particularly valuable when there are a limited number of studies and important decisions are being made globally about the treatment of children with growth faltering under 6 months of age based on them. Additionally, this section was very valuable to the World Health Organization in their guidance development. We presented these same results to the WHO Guideline Development Group (GDG) led by world-leading experts and our findings, including the methodological aspects of our findings, were extensively discussed by the committee. As we mentioned, the committee found our Results easy to follow and very relevant and useful, and they were ultimately incorporated into the recent WHO guidance on the prevention and management of wasting and nutritional oedema (acute malnutrition) in infants and children under 5 years released in June 2023.

2.Please present results based on the specific objectives of the study. To me, the results are not clear. They are more of methodological explanations rather than reporting was found out in line with the study objectives. Short of this, the reader gets lost because results look like extended sections of methodology section.

We have a subsection called “Outcomes of Interest”. Once again, the careful description of methodological issues is strongly recommended as they are critical to interpreting the findings of the review.

3.Actually, the results writeup should be more than the methodology section.

Please refer to our response in point #1 above. We do offer a discussion of the studies’ findings in addition to the discussion of their methodological approaches, which is essential to their interpretation.

4.Organise your manuscript according to the journal guidelines. In most cases, results are followed by discussion of results, Conclusion and recommendations.

We have done so. The journal allows Results, Discussion and Conclusion, as well as subsections within.

5. Create a subheading that clearly shows the limitations of the study.

Thank you. We have created a subheading.

6. This table is not readable. Please use a clear table and if not readable, why include it?

We have been specifically instructed by the editorial assistant to convert all of our tables from PDF to TIFF format and we have followed their request. The reviewer should be able to access them by downloading them and they are not blurry in that format. Unfortunately, by design of the TIFF format, they do not load the same in a combined PDF (we believe the reviewer can download them from the reviewer site). We ask the reviewer to please refer to the figures we have submitted initially and/or consult the editorial office for assistance as this is not within our control.

7. See additional comments in the attached copy.

The additional comments concern readability of the Figures and Tables and organization, which we have addressed above. We have corrected all other citation requests in the attachment.

**Reviewer #3:** The manuscript has been revised well. I hope this manuscript will be
acceptable.

We thank the Reviewer and appreciate their kind comments.
